# Supplementary material for: Causes and Consequences of A Glutamine Induced Normoxic HIF1 Activity for the Tumor Metabolism
Source: Int J Mol Sci. 2019 Sep 24;20(19):4742. doi: 10.3390/ijms20194742 (PMC6802203; doi:10.3390/ijms20194742)
Supplement: Supplementary file 1 [file ijms-20-04742-s001.pdf]

Supplemental Figures

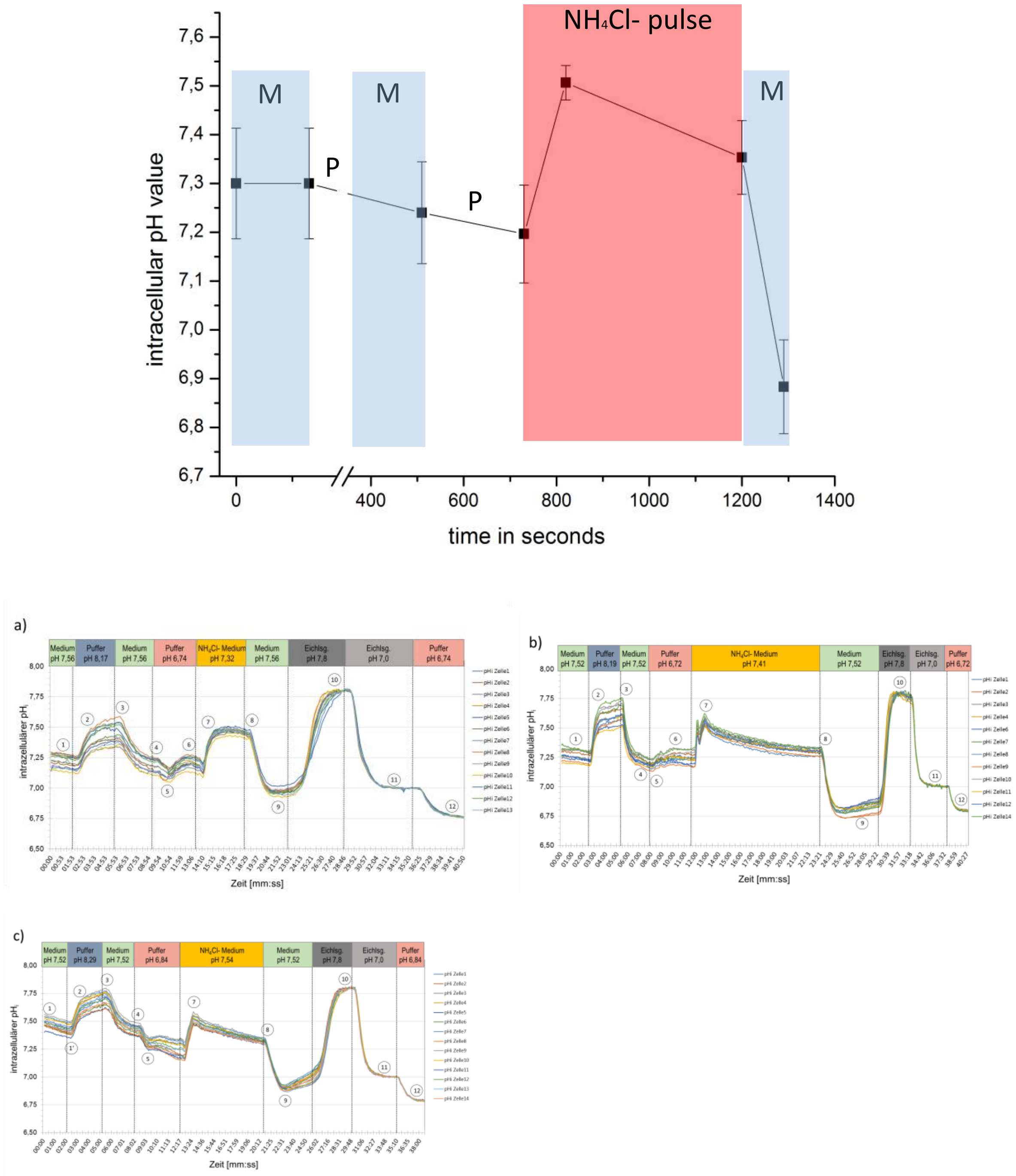

Supplemental Figure S1. Measurement of the intracellular pH value after ammonium chloride impulse The scheme shows the average of three independent experiments, each with >13 parallel measured cells of the MDA-MD-231 cell line. The intracellular pH value of the cells in the RPMI 1640 medium (M) (pH value of the medium was 7.53 ± 0.02 (SD)) used was 7.28 ± 0.10. A pulse of 5 mM NH_4Cl (in red) (pH value of RPMI medium with NH_4Cl was 7.42 ± 0.11) which contains by calculation a concentration of 168 ± 42 µM ammonia gas in the RPMI medium (aqueous solution) was given. Within about 90 s after the NH_4Cl-pulse, the intracellular pH value of the cells rapidly increased for

0.23 pH units significantly to a maximum pH value of 7.51 ± 0.04 (p = 0.035, Student t-test). After reaching this maximum, the intracellular pH value of the cells decreased to a pH value of 7.35 ± 0.08 in the next few minutes to find a new equilibrium. Then the RPMI medium containing 5 mM NH_4Cl (ammonia gas dissolved at 168 µM) was replaced with RPMI medium **(M)** without NH_4Cl. The intracellular pH value of the cells decreased rapidly to a pH value of 6.89 ± 0.10 within 90 s of use of the initial RPMI medium **(M)**. This correspond to a significant decrease in the intracellular pH value of the cells in RPMI medium **(M)** in comparison to the starting point of about 0.39 pH units (p = 0.008, Student t-test). (**P)** Represent different buffer systems with different pH values to test the viability of the investigated cells. (**a**–**c** represent the original data of three independent experiments each with > 10 cells).

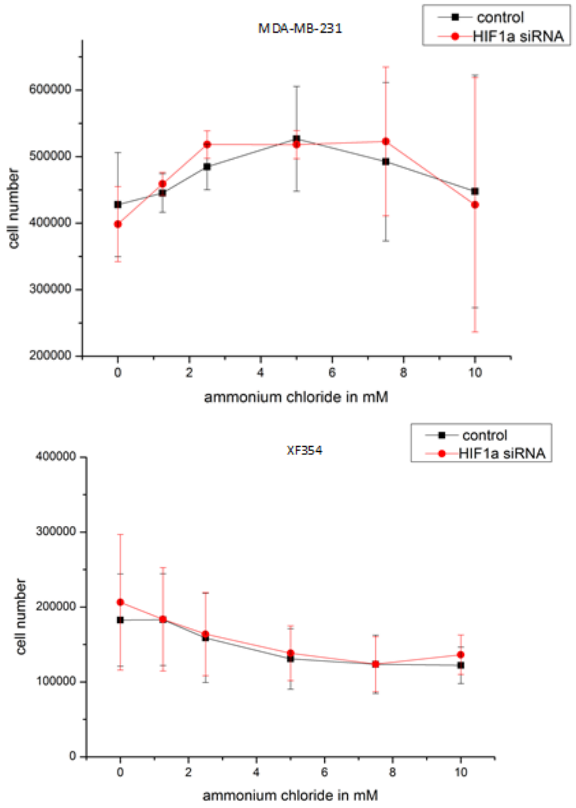

Supplemental Figure S2. Analysis of the potential of ammonium/ammonia to replace glutamine as nitrogen source in term of proliferation. 72 h after the onset of the experiment using concentration of 0–10 NH_4CL without the application of additional glutamine (using medium without glutamine and 11.1 mM glucose with a pH value of 7.3–7.6) no significant proliferation was detected in MDA-MB-231 and XF354 cells. The experiment starts with a cell number of 200,00 cells. At higher level of NH_4CL >5 mM, the cell numbers especially in the cell line XF354 are further reduced. After a proliferation time of 72 h the cell number in MDA-MB-231 cell fluctuate between 400,000 to 500,000 cells whereas the cell number in XF354 cell was between 200.000 and 100.000 cells. The knock down of HIF1α do not had an effect. The data were performed from at least three independent experiments.

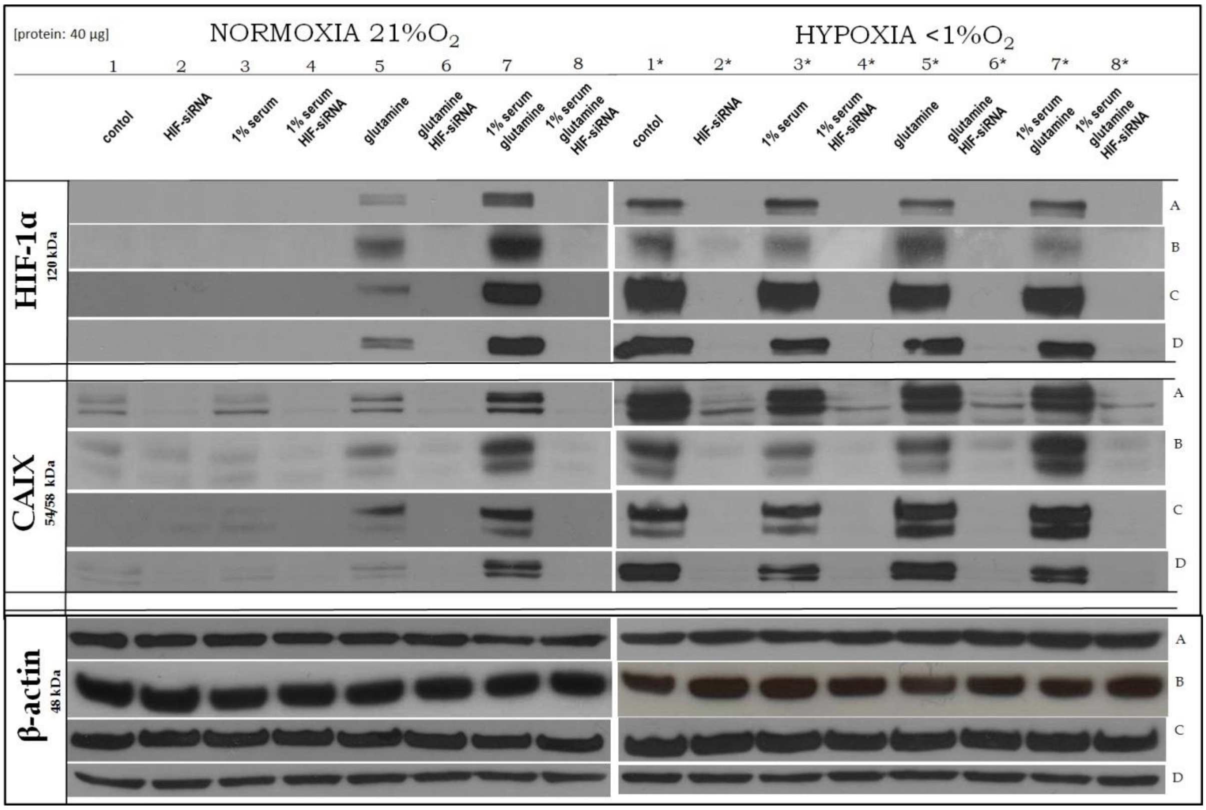
[protein: 40 µg]
NORMOXIA 21%O2
HYPOXIA <1%O2
1 2 3 4 5 6 7 8
1* 2* 3* 4* 5* 6* 7* 8*
contol
HIF-siRNA
1% serum
1% serum HIF-siRNA
glutamine
glutamine HIF-siRNA
1% serum glutamine
1% serum glutamine HIF-siRNA
HIF-1α
120 kDa
CAIX
54/58 kDa
β-actin
48 kDa
A
B
C
D

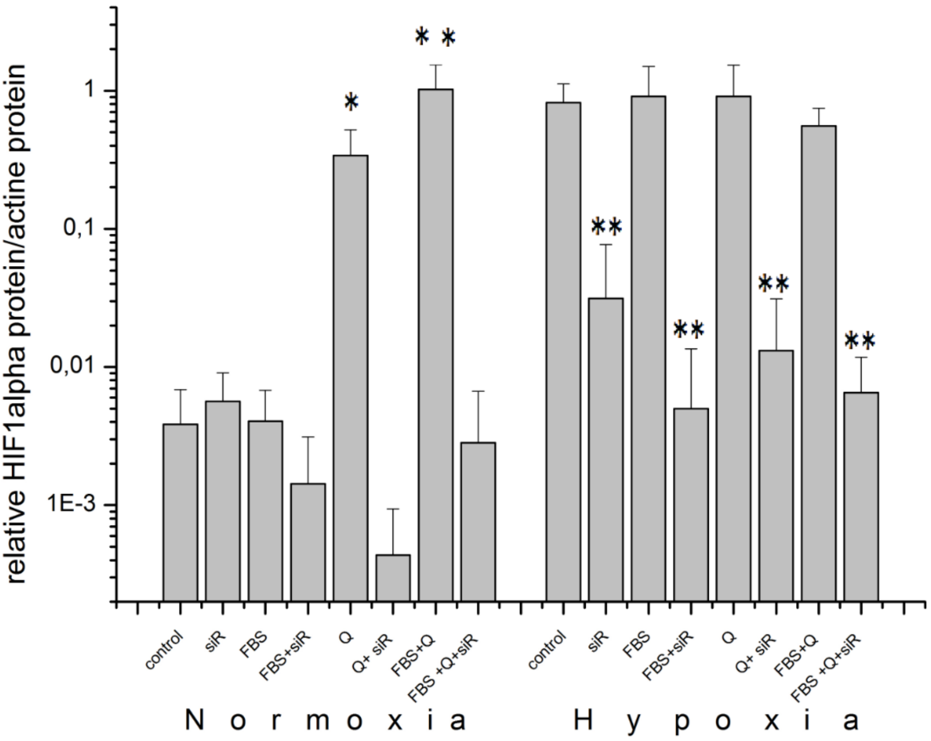
relative HIF1alpha protein/actine protein
1
0,1
0,01
1E-3
control
siR
FBS
FBS+siR
Q
Q+ siR
FBS+Q
FBS +Q+siR
N o r m o x i a
H y p o x i a

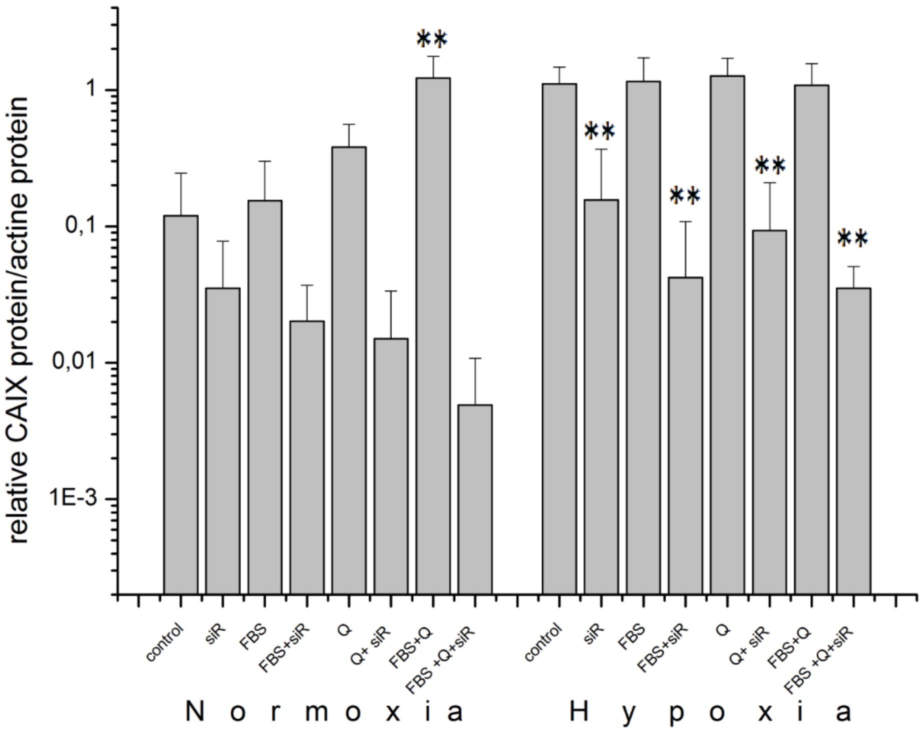

Supplemental Figure S3. Western blot analysis of HIF1α, CAIX, and β-actin levels after the application of different treatments. (Lane 1) Medium control, (lane 2) siRNA-treated against HIF1α, (lane 3) 1% fetal bovine serum, (lane 4) 1% fetal bovine serum and siRNA-treated against HIF1α, (lane 5) 5 mM glutamine, (lane 6) 5 mM glutamine and siRNA-treated against HIF1α, (lane 7) 1% fetal bovine serum and 5 mM glutamine, and (lane 8) 1% fetal bovine serum (FBS) and 5 mM glutamine (Q) and siRNA-treated against HIF1α (siR) under normoxic or hypoxic (*) conditions in the cell line MDA-MB-231 in four independent experiments (**A**–**D**). Twenty-four hours after the start of the tests. Experiments were performed in RPMI 1640 without glutamine with 11.1 mM glucose. Please, see also the densitometric evaluation of the Western Blots (* $-p < 0.05$; ** $-p < 0.01$, Student *t*-test compared to control).

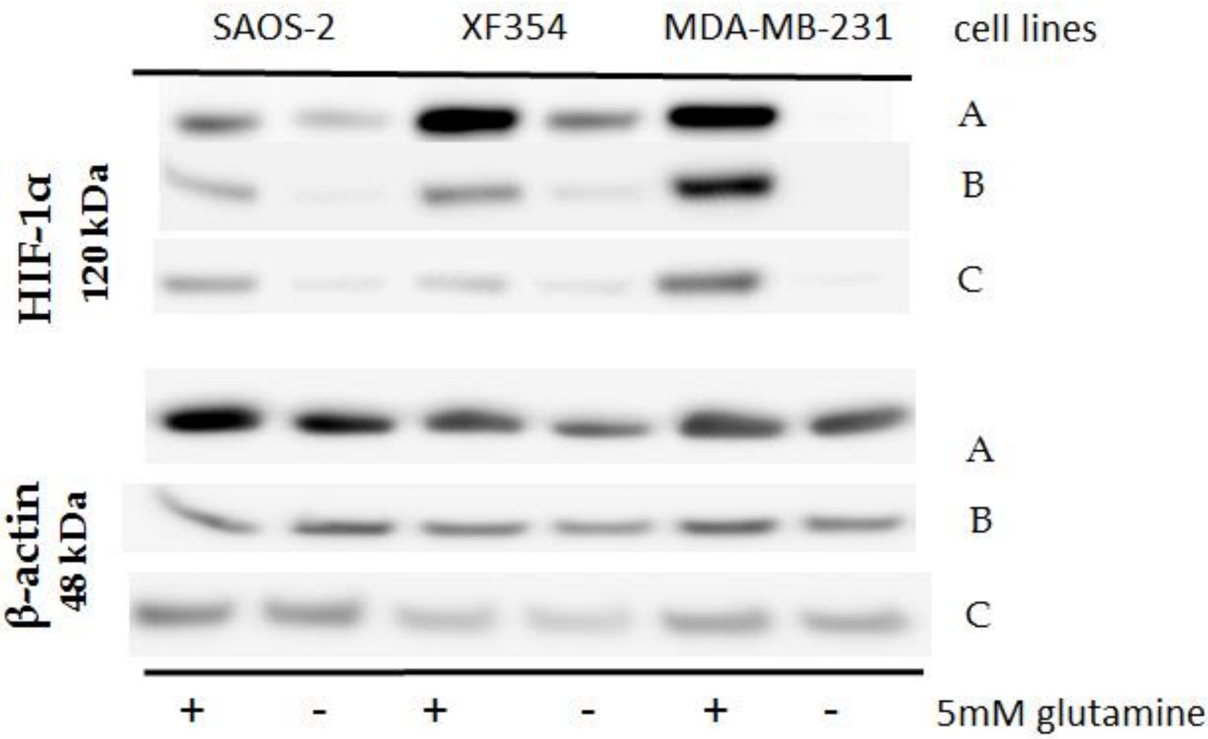

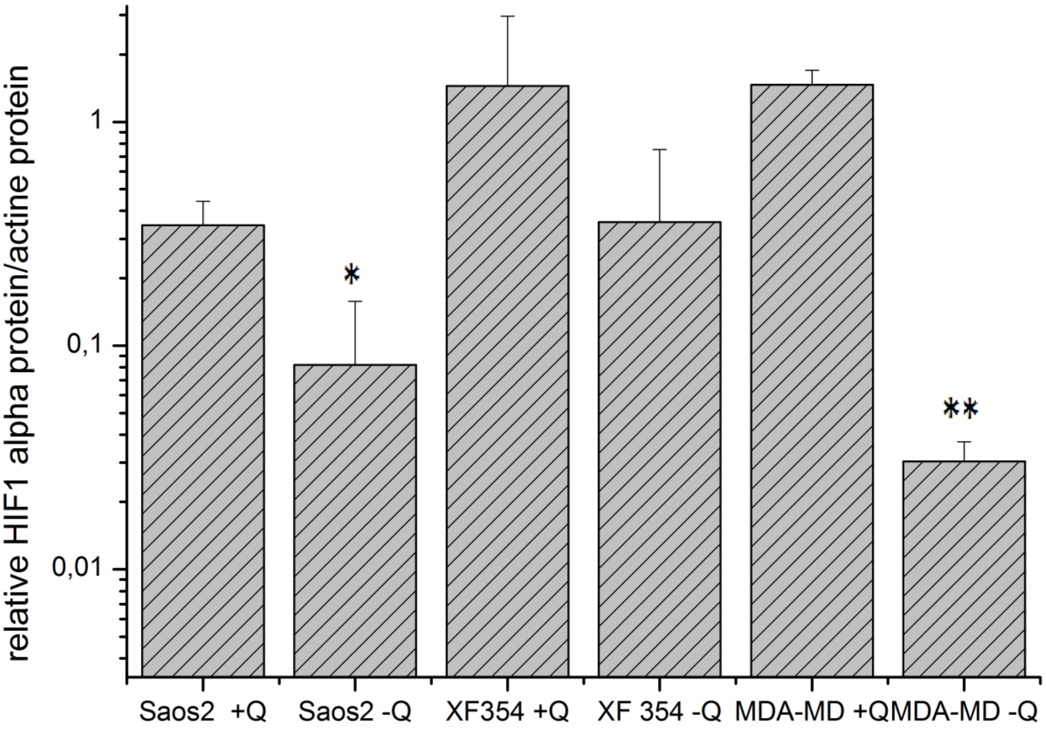

Supplemental Figure S4. Western blot analysis of HIF1*α* and β-actin levels after the application of glutamine in different cell lines. Saos-2, XF354, and MDA-MB-231 cells were treated with 5 mM glutamine (+Q) using RPMI medium without glutamine (−Q) under normoxic condition for 24 h. Experiments were performed in RPMI 1640 without glutamine with 11.1 mM glucose in three independent experiments. Please, see also the densitometric evaluation of the Western Blots (* $-p <$ 0.05; ** $-p < 0.01$, Student *t*-test compared to control).

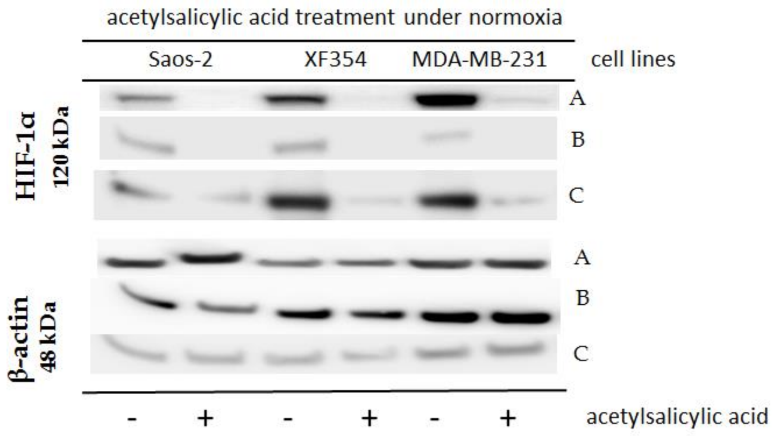

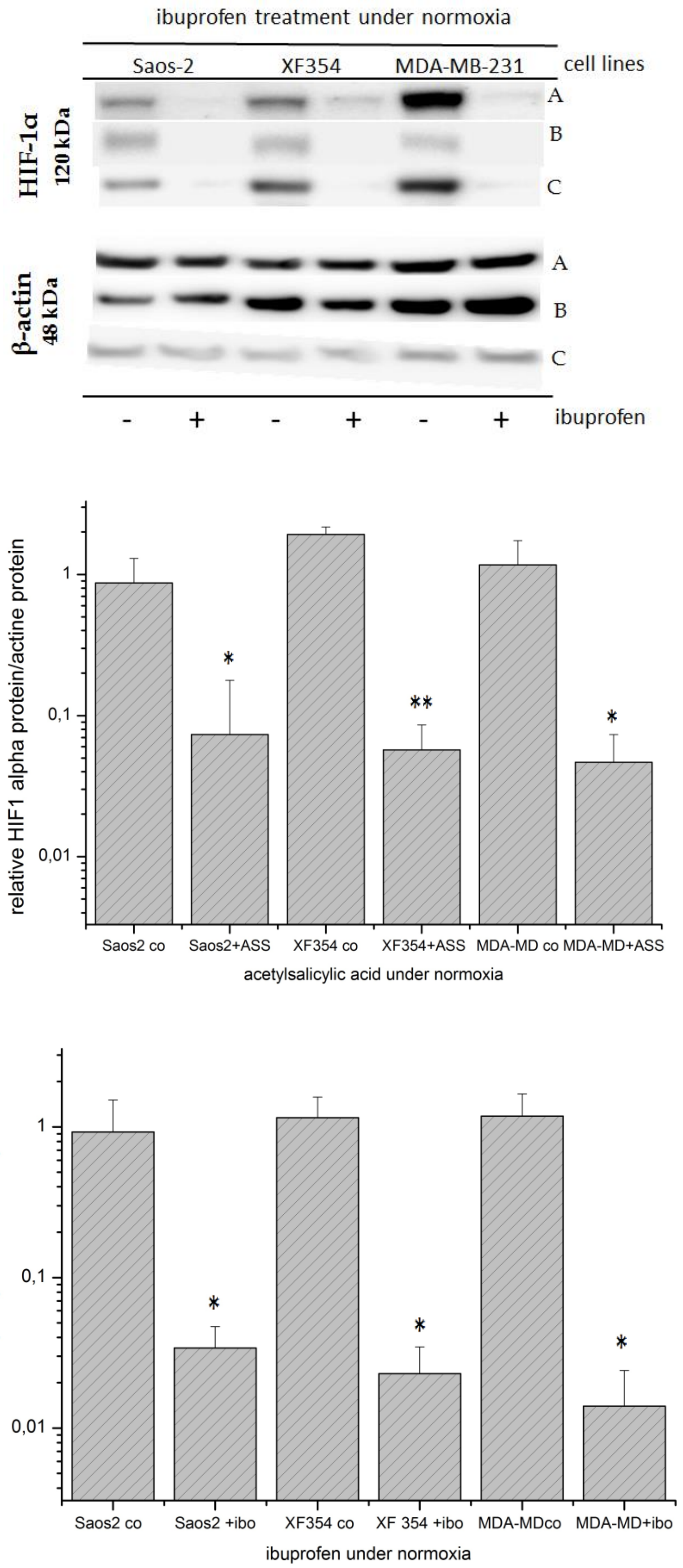

Supplemental Figure S5. Western blot analysis of HIF1α and β-actin levels after the application of 10 mM acetylsalicylic acid (ASS) or 3 mM ibuprofen (ibo) in different cell lines (Saos-2, XF354, and MDA-MB-231) under normoxia. Acetylsalicylic acid or ibuprofen can reduce the glutamine induced

normoxic HIF1 α-level. Experiments were performed in RPMI 1640 with 5 mM glutamine and with 11.1 mM glucose under normoxic condition for 24 h in three independent experiments. Please, see also the densitometric evaluation of the Western Blots (* $-p < 0.05$; ** $-p < 0.01$, Student t-test compared to control (co)).

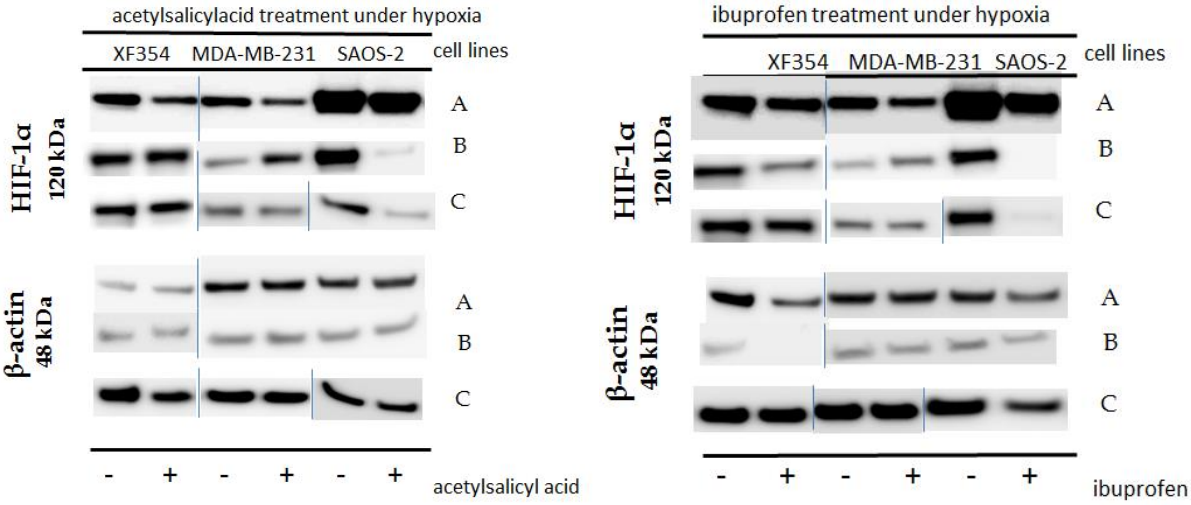

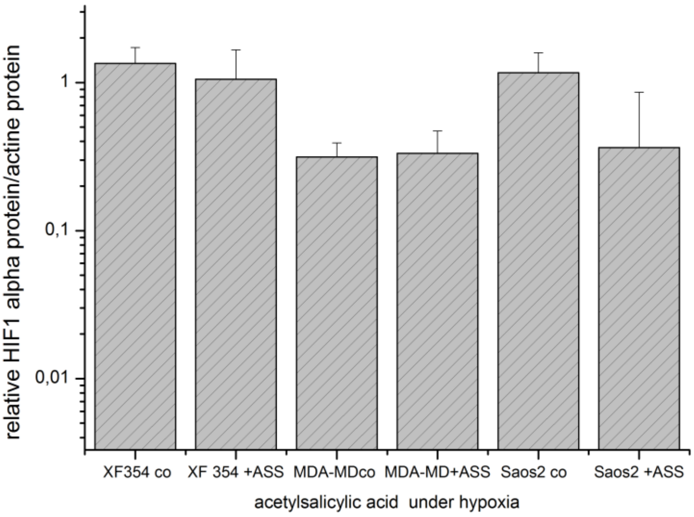

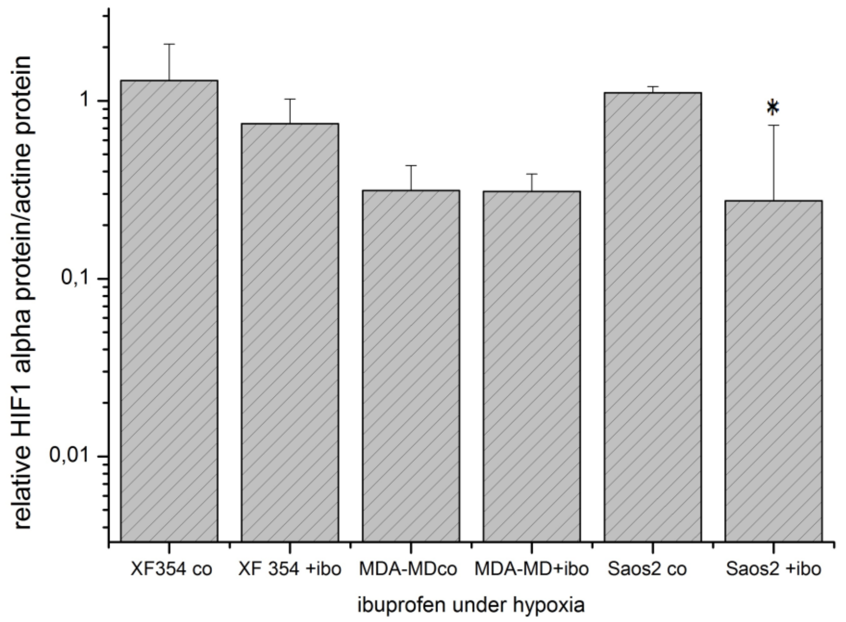

Supplemental Figure S6. Western blot analysis of HIF1α and β-actin levels after the application of 10 mM acetylsalicylic acid (ASS) or 3 mM ibuprofen (ibo) in different cell lines (Saos-2, XF354, and MDA-MB-231) under hypoxia. Experiments were performed in RPMI 1640 with 5 mM glutamine and with 11.1 mM glucose under hypoxic condition (oxygen >0.1%) for 24 h in three independent experiments. Please, see also the densitometric evaluation of the Western Blots (* −$p < 0.05$, Student t-test compared to control (co)).

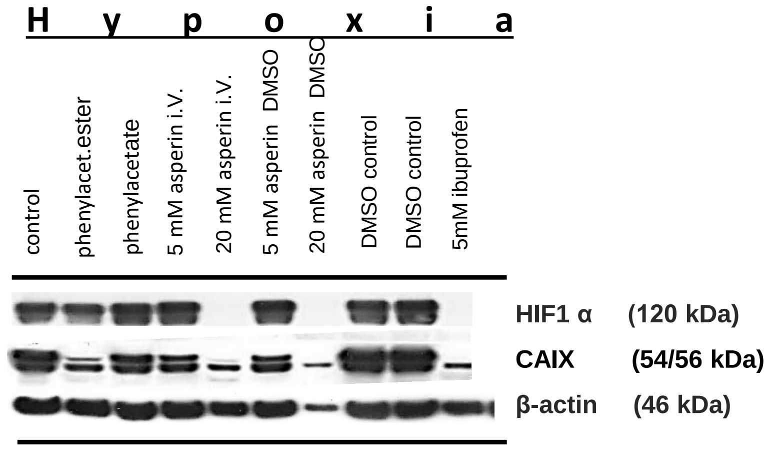

Supplemental Figure S7. Western blot analysis of HIF1α, CAIX and β-actin expression after the application of different concentrations of aspirin or phenylacetate ester, phenylacetate, ibuprofen as further controls in the MCF-7 cells. The DMSO controls (line 8 and 9) represent the quantity of DMSO that was given in line 6 and 7. All experiments were performed using RPMI 1640 medium supplemented (with 2.2 mM glutamine and 10% serum under hypoxic (0.1% oxygen) conditions after 24 h.

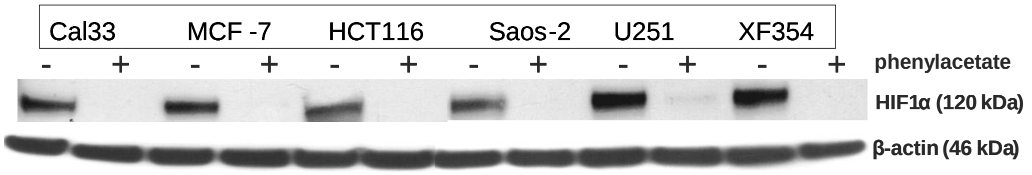

Supplemental Figure S8. Western blot analysis of HIF1α and β-actin w/o the application of glucose (20 mM), phenylacetate (10 mM), and benzoate (10 mM) (this solution is similar to a treatment, which

is given for treatment of hyperammonemia known as AMMONUL®) in the Cal33 (lanes 1–2), MCF-7 (lanes 3–4), HCT116 (lanes 5–6), Saos-2 (lanes 7–8), U251 (lanes 9–10) and XF354 (lanes 11–12) cell lines. Please note the suppression of HIF1α expression is a response to the application of glucose, phenylacetate and benzoate under hypoxic conditions. Experiments were performed in RPMI 1640 medium with serum supplementation under hypoxic conditions. Cells were harvested 3 h after the application of these substances.

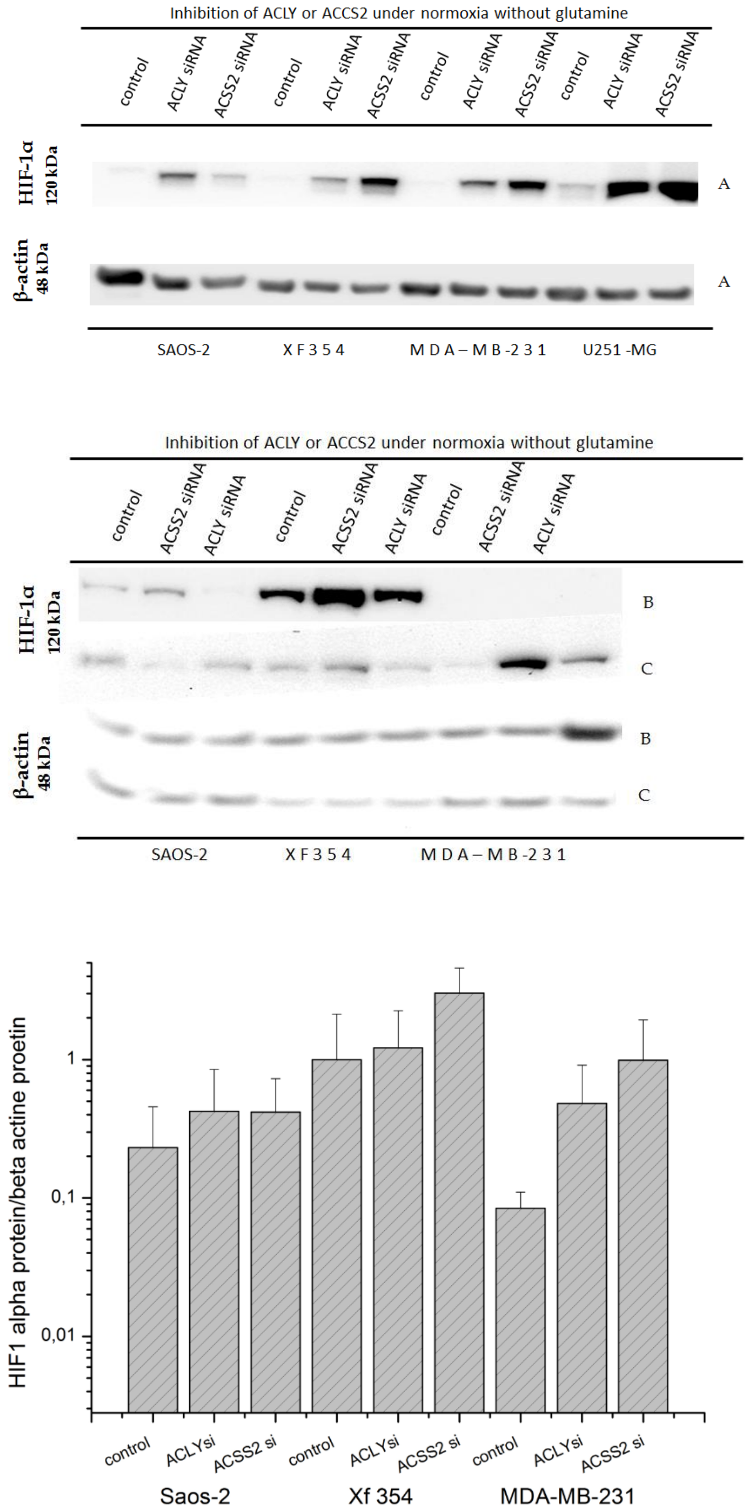

Supplemental Figure S9a. Western blot analysis of HIF1α and β-actin levels after the application of siRNA against ATP citrate lyase (ACLY) or the cytosolic form of the acetyl-CoA synthetase (ACSS2) in cells of different lineages. Saos-2, XF354 andMDA-MB-231 cells were treated with 20 nM siRNA

against ACSS2 or ACLY in RPMI 1640 medium without glutamine under normoxic conditions for 48 h in three independent experiments. Please, see also the densitometric evaluation of the Western Blots.

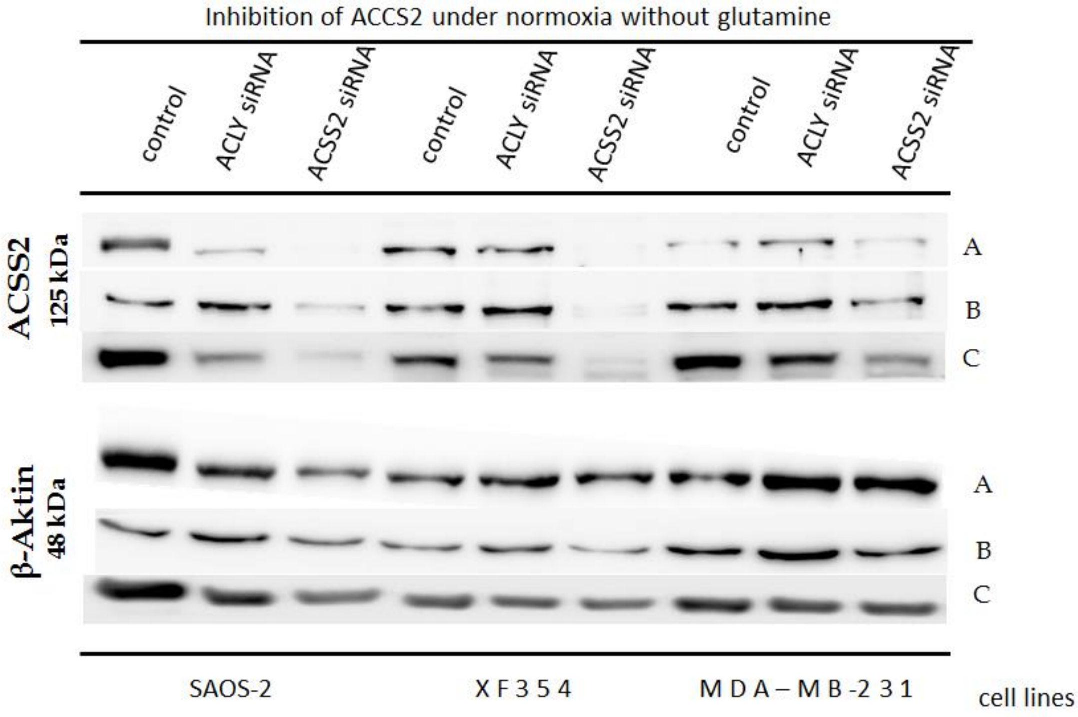

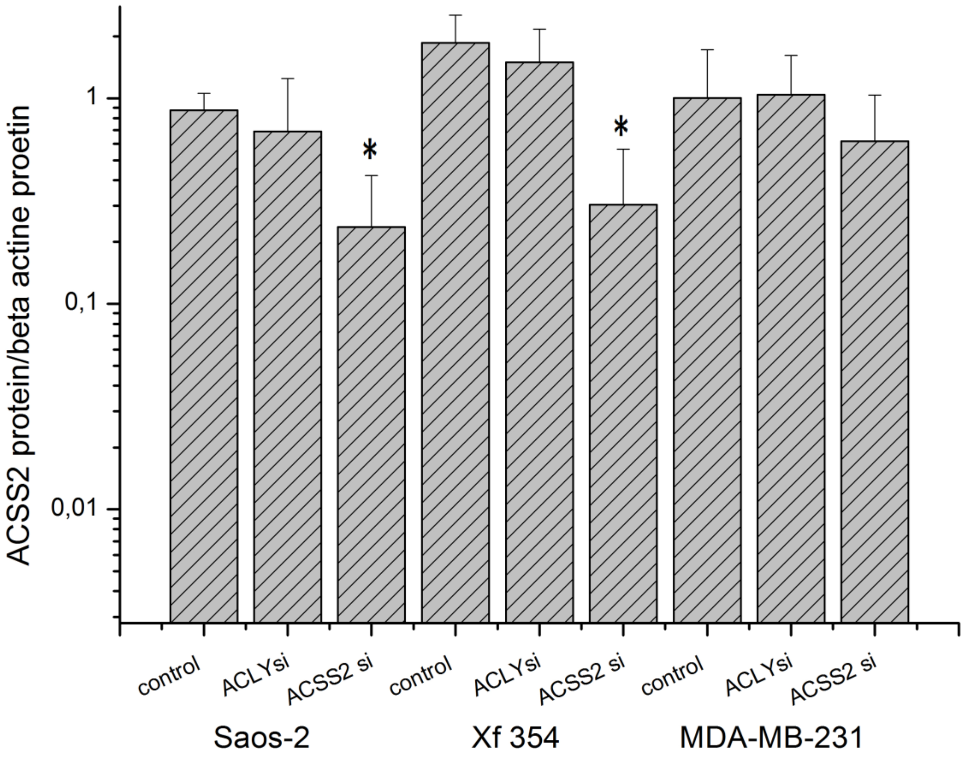

Supplemental Figure S9b. Western blot analysis of ACSS2 and β-actin levels after the application of siRNA against ATP citrate lyase (ACLY) or the cytosolic form of the acetyl-CoA synthetase (ACSS2) in cells of different lineages. Saos-2, XF354 and MDA-MB-231 cells were treated with 20 nM siRNA against ACSS2 or ACLY in RPMI medium without glutamine under normoxic conditions for 48 h in three independent experiments. Please, see also the densitometric evaluation of the Western Blots.

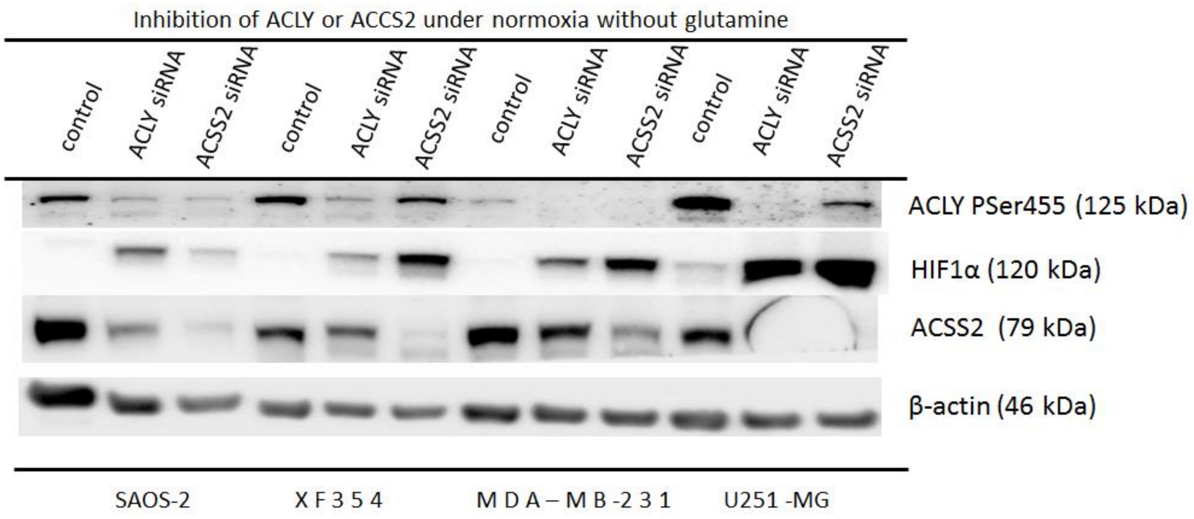

Supplemental Figure S9c. Western blot analysis of HIF1α, β-actin, ACLY (Ser 455) and ACSS2 levels after the application of siRNA against ATP citrate lyase (ACLY) or the cytosolic form of the acetyl-CoA synthetase (ACSS2) in cells of different lineages. Saos-2, XF354, MDA-MB-231 and U251-MG cells were treated with 20 nM siRNA against ACSS2 or ACLY in RPMI medium without glutamine under normoxic conditions for 48 h.

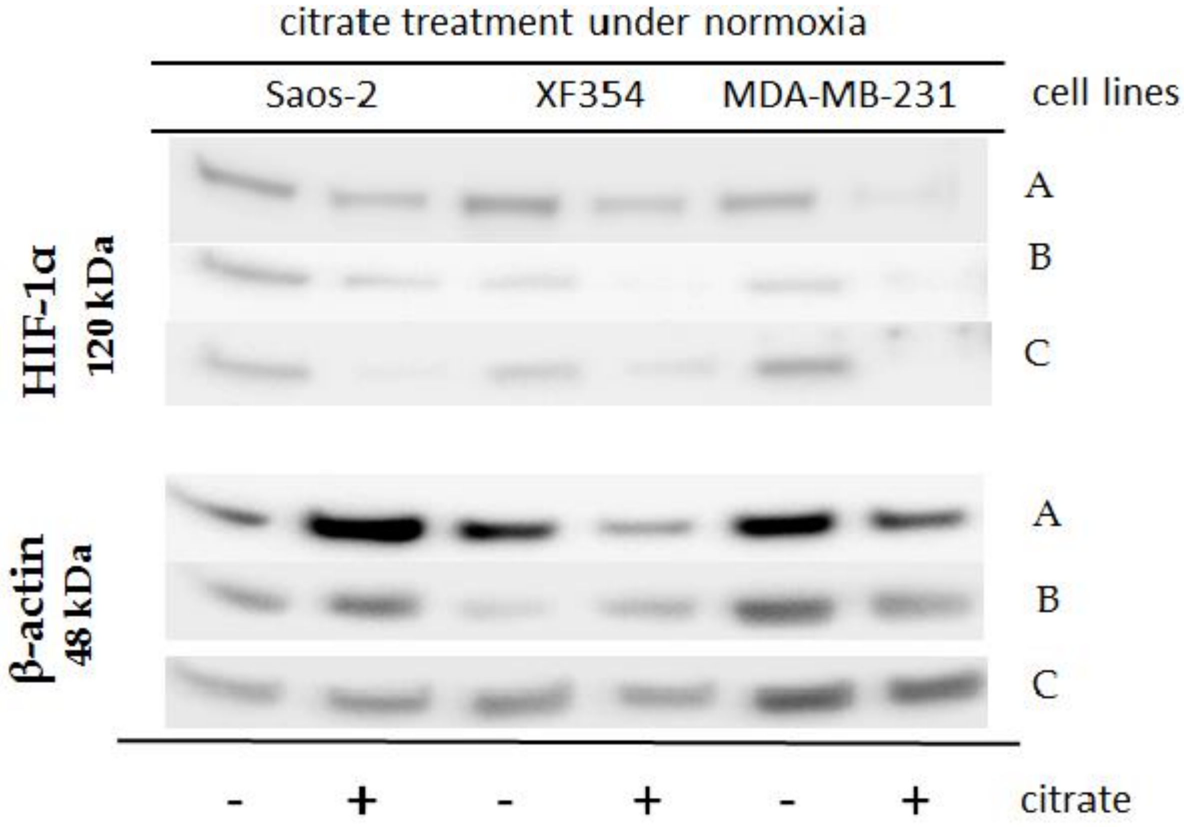

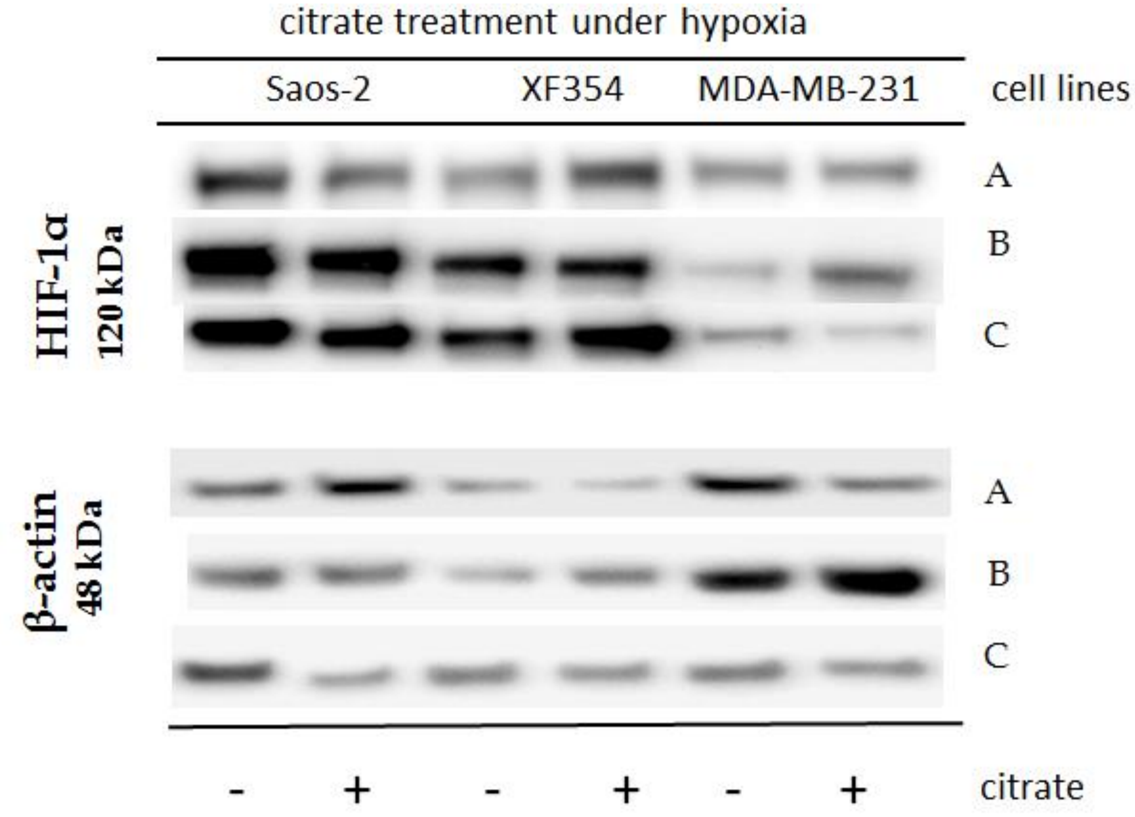
citrate treatment under hypoxia
Saos-2
XF354
MDA-MB-231
cell lines
HIF-1α
120 kDa
β-actin
48 kDa
A
B
C
A
B
C
- + - + - +
citrate

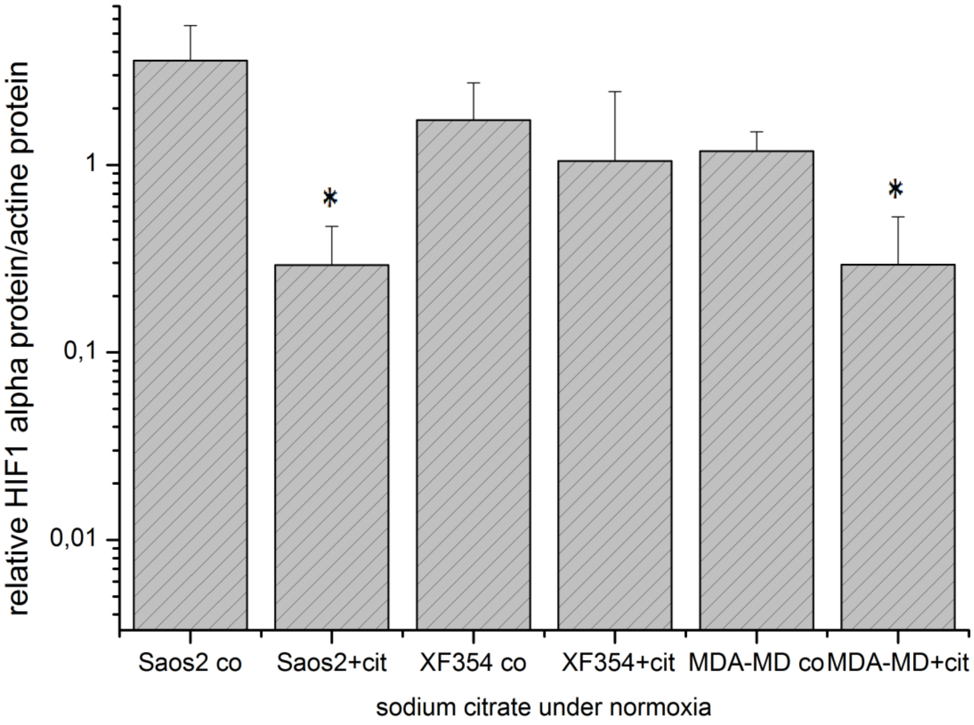
relative HIF1 alpha protein/actine protein
1
0,1
0,01
*
*
Saos2 co
Saos2+cit
XF354 co
XF354+cit
MDA-MD co
MDA-MD+cit
sodium citrate under normoxia

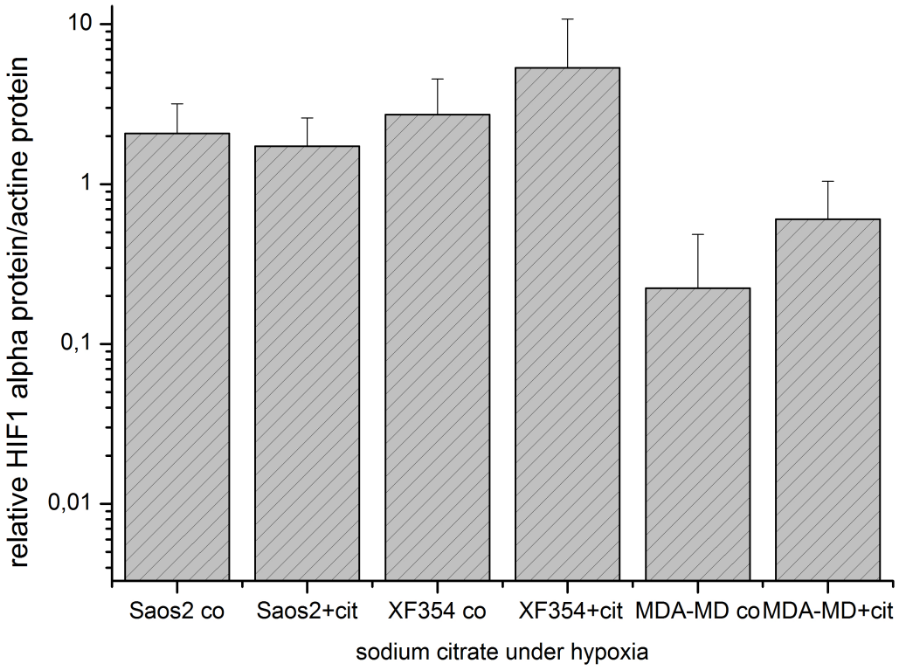

Supplemental Figure S10. Western blot analysis of HIF1α and β-actin levels after the application of 8mM sodium citrate (cit) in cell lines Saos-2 and MDA-MB-231 and 4mM sodium citrate in cell line XF354 under normoxia or hypoxia. Experiments were performed in RPMI 1640 with 5 mM glutamine and with 11.1 mM glucose for 24 h in three independent experiments. Please, see also the densitometric evaluation of the Western Blots (* −$p < 0.05$ Student t-test compared to control (co)).

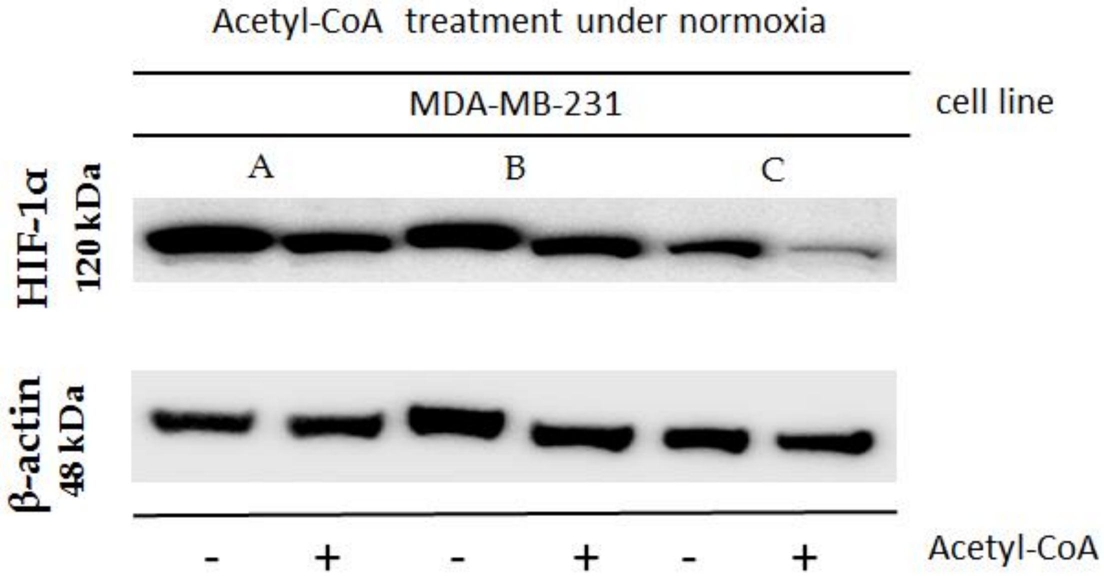

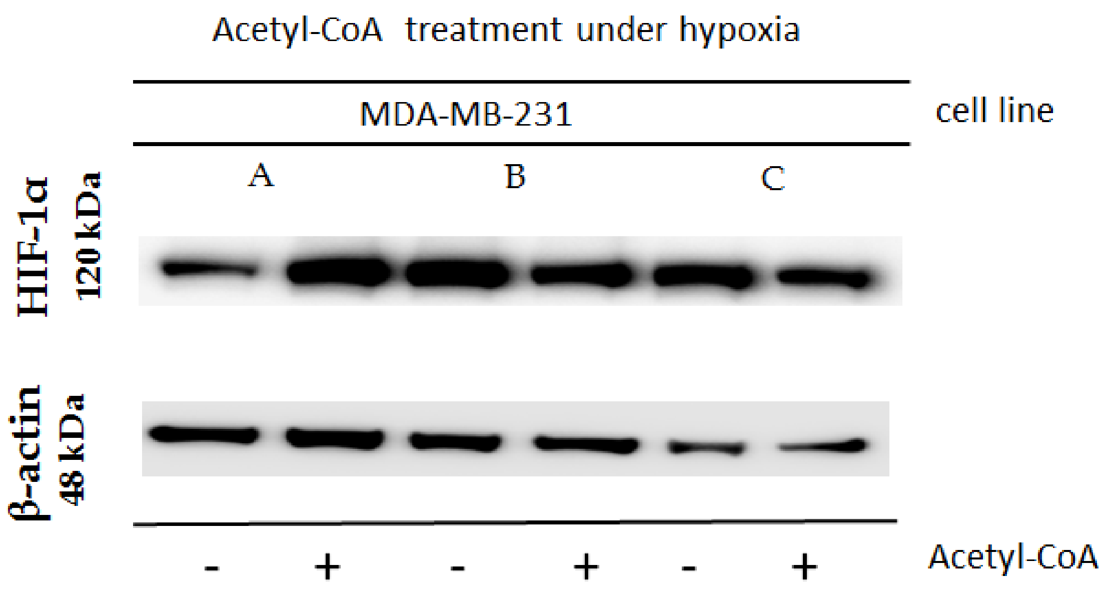

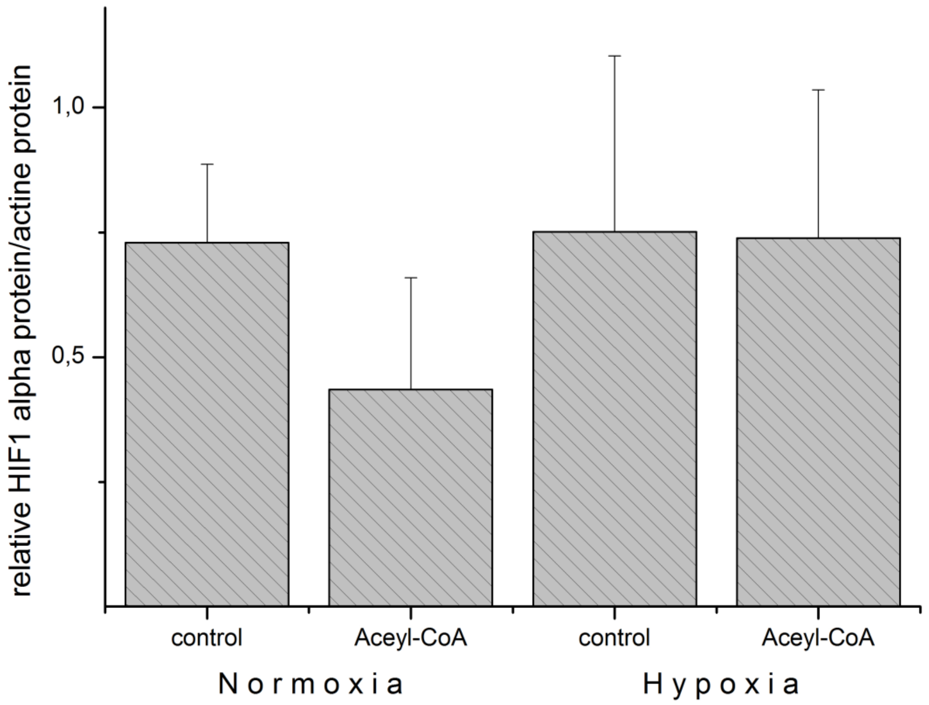

Supplemental Figure S11. Western blot analysis of HIF1*α* and β-actin levels after the application of 8 mM acetyl-CoA in cell line MDA-MB-231 under normoxia or hypoxia. Experiments were performed in RPMI 1640 with 5 mM glutamine and with 11.1 mM glucose for 12 h in three independent experiments. Please, see also the densitometric evaluation of the Western Blots (for the normoxic conditions a $p = 0.13$ was calculated by Student *t*-test compared to control).

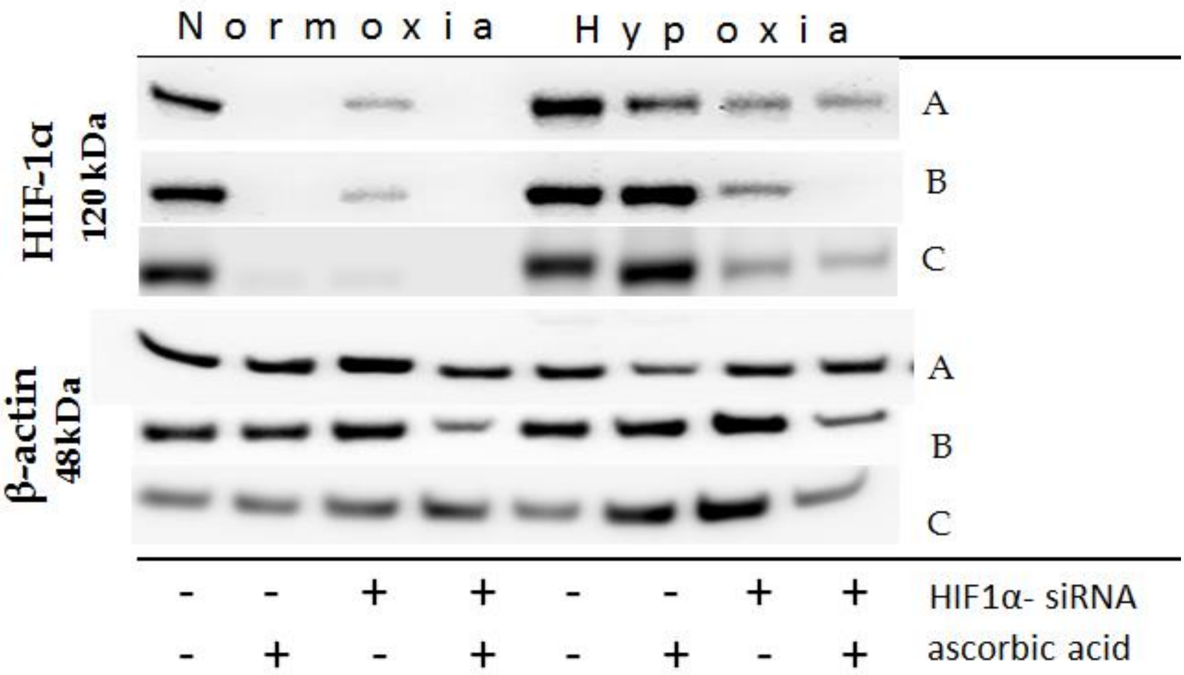

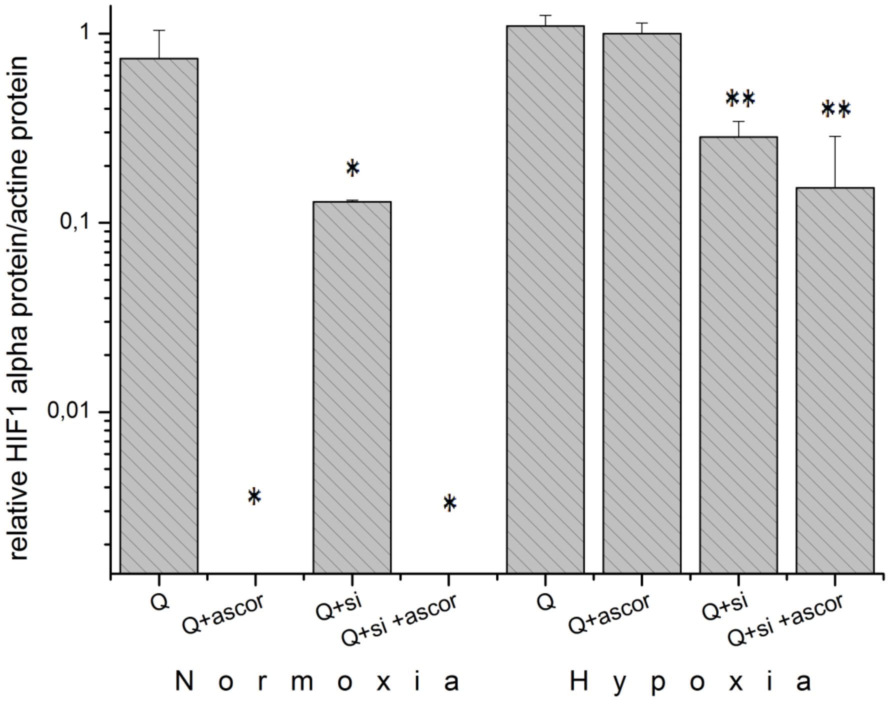

Supplemental Figure S12. Western blot analysis of HIF1α and β-actin levels after the application of 100µM ascorbic acid (ascor) in cell line MDA-MB-231 under normoxia or hypoxia. Experiments were performed in RPMI 1640 with 5 mM glutamine and with 11.1 mM glucose for 24 h in three independent experiments. Please, see also the densitometric evaluation of the Western Blots (* $-p < 0.05$; ** $-p < 0.01$, Student *t*-test compared to control (Q)).

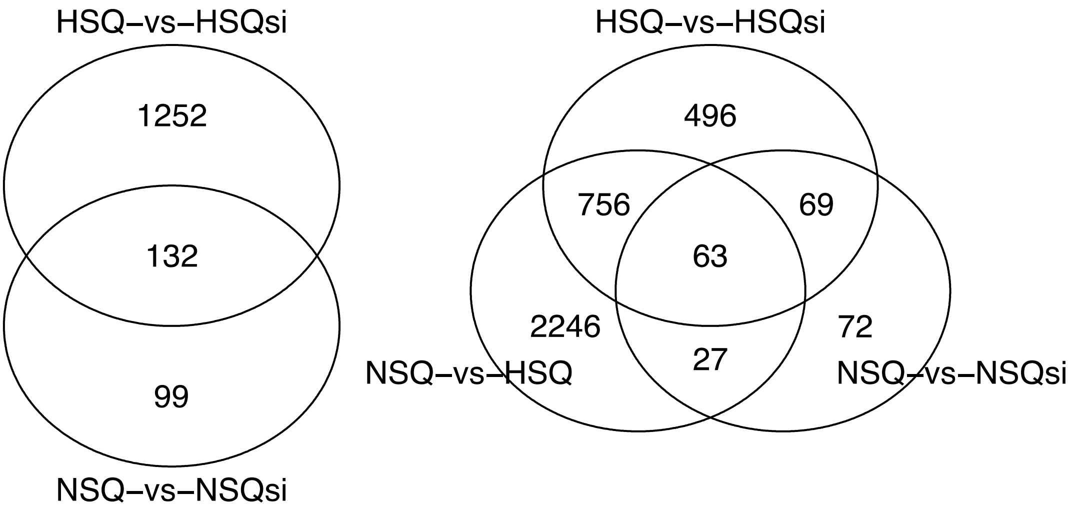

Supplemental Figure S13. Venn diagram of analysed samples corresponding to Figure 1, 24 h after the treatment. Four independent experiments were analysed via deep sequencing analyses and the number of differentially expressed genes are compared by Venn diagram. Normoxic samples treated with 1% fetal bovine serum and 5 mM glutamine (NSQ) were blotted against normoxic samples treated with 1% fetal bovine serum, 5 mM glutamine and siRNA-treated against HIF1α (NSQsi) and against hypoxic samples treated with 1% fetal bovine serum and 5 mM glutamine (HSQ) were blotted against hypoxic samples treated with 1% fetal bovine serum, 5 mM glutamine and siRNA-treated against HIF1α (HSQsi). 132 genes were differentially expressed by HIF1 under both normoxic and hypoxic conditions.

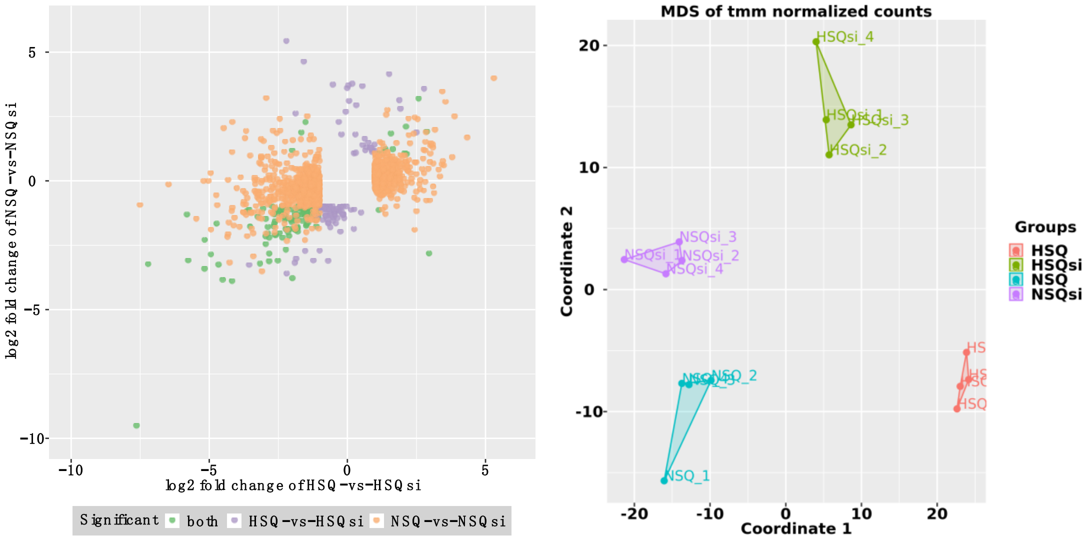

Supplemental Figure S14. Scatterplot and MDS plot of the same samples, which were treated and analysis in Supplemental Figure S3. The scatterplot of the logarithmic fold changes demonstrated the quality and quantity of the differentially expressed genes by normoxic or hypoxic cells treated with or without HIF1α siRNAs. The MDS plot shows the tmm-normalized gene expression of all four different treatments for the four independent experiments.

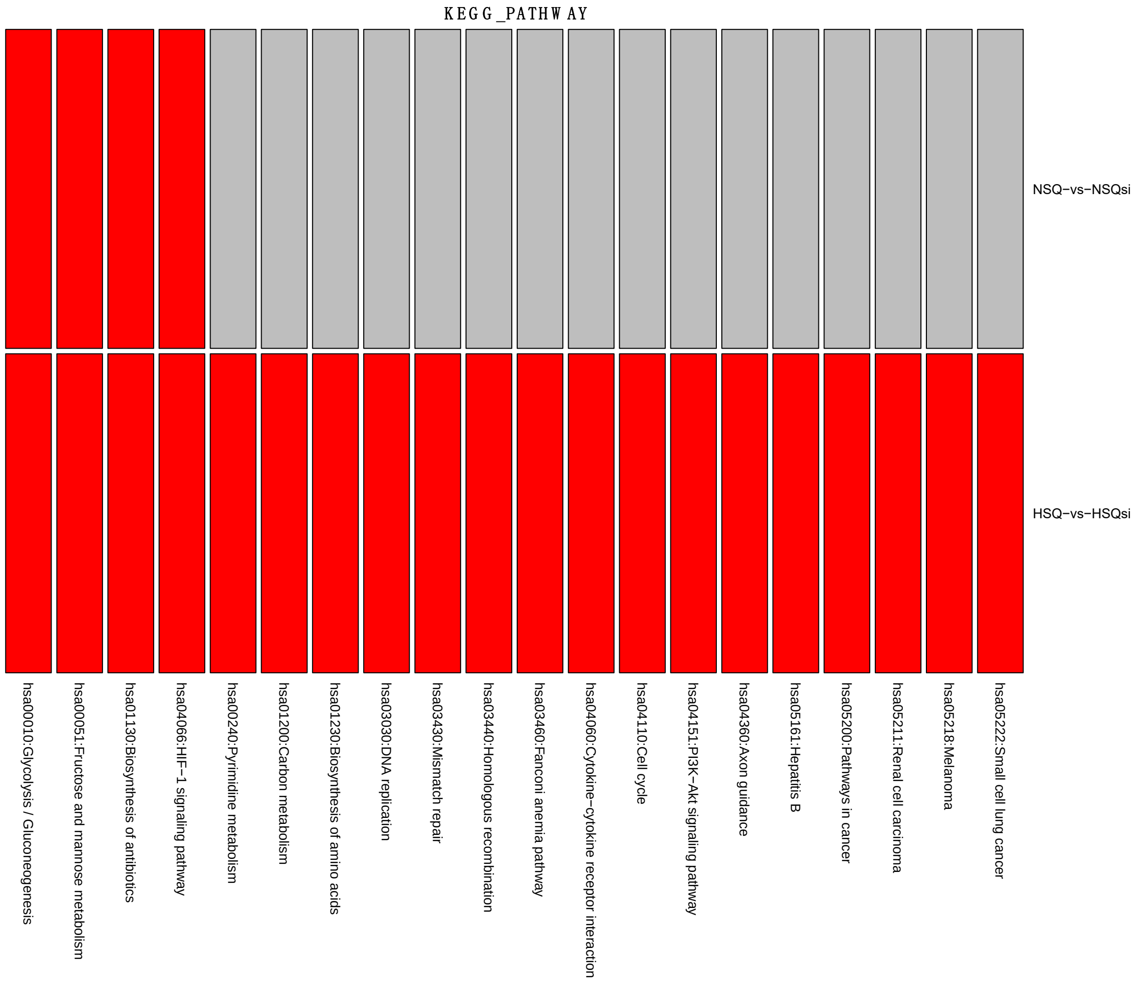

Supplemental Figure S15. KEGG pathway analysis, which demonstrated that HIF1 affected glycolysis and carbon metabolism by analysis the effects of 1% fetal bovine serum and 5 mM glutamine (NSQ or HSQ) against treatment with 1% fetal bovine serum, 5 mM glutamine and siRNA-treated against HIF1α (NSQsi or HSQsi) under both hypoxic and normoxic conditions. Red corresponds to significant regulated KEGG pathways.

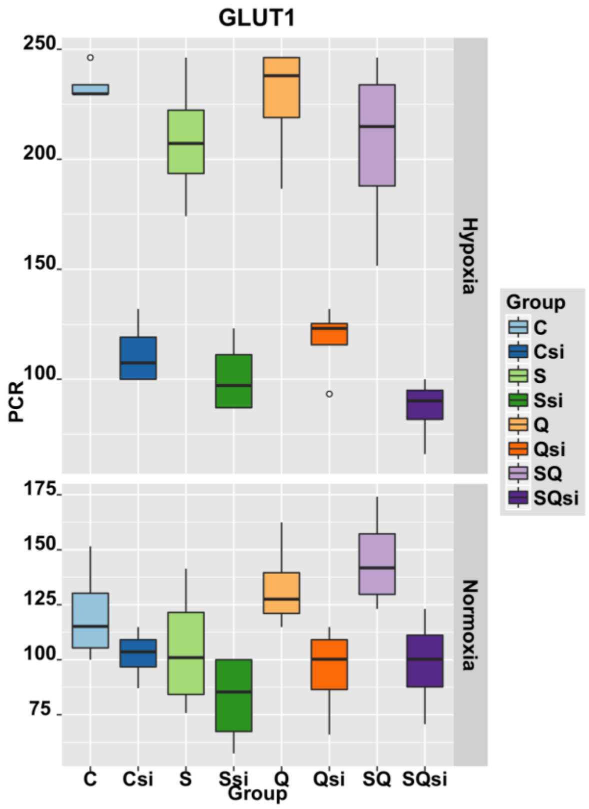
GLUT1
PCR
250
200
150
100
175
150
125
100
75
Hypoxia
Normoxia
C Csi S Ssi Q Qsi SQ SQsi
Group
Group
C
Csi
S
Ssi
Q
Qsi
SQ
SQsi

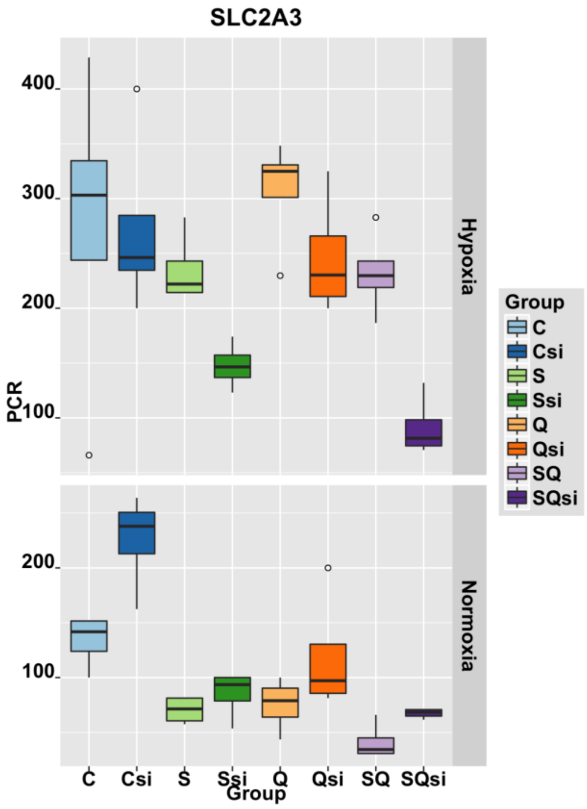
SLC2A3
PCR
400
300
200
100
200
100
Hypoxia
Normoxia
C Csi S Ssi Q Qsi SQ SQsi
Group
Group
C
Csi
S
Ssi
Q
Qsi
SQ
SQsi

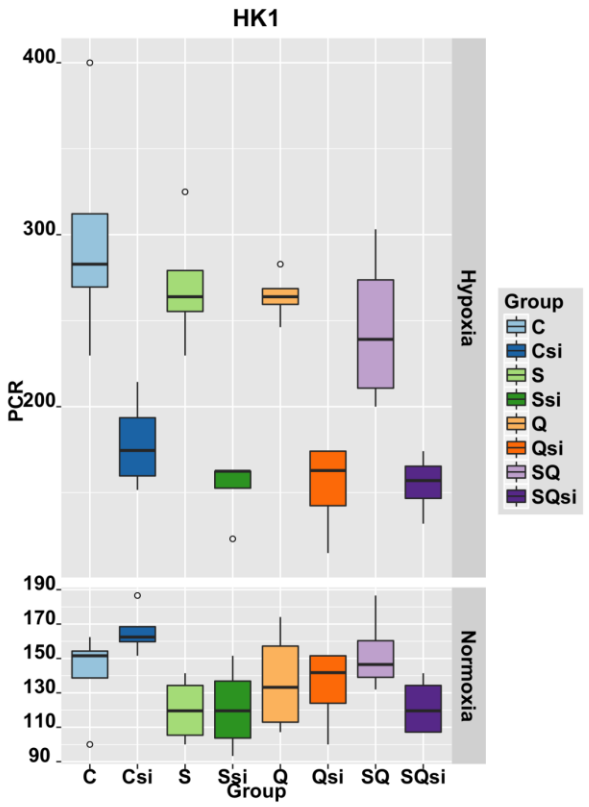
HK1
PCR
400
300
200
190
170
150
130
110
90
Hypoxia
Normoxia
C Csi S Ssi Q Qsi SQ SQsi
Group
Group
C
Csi
S
Ssi
Q
Qsi
SQ
SQsi

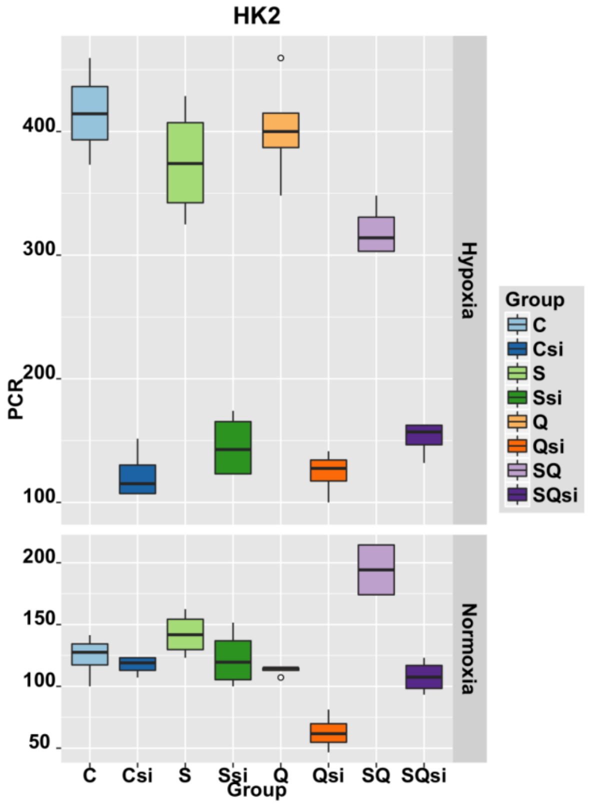
HK2
PCR
400
300
200
100
200
150
100
50
Hypoxia
Normoxia
C Csi S Ssi Q Qsi SQ SQsi
Group
Group
C
Csi
S
Ssi
Q
Qsi
SQ
SQsi

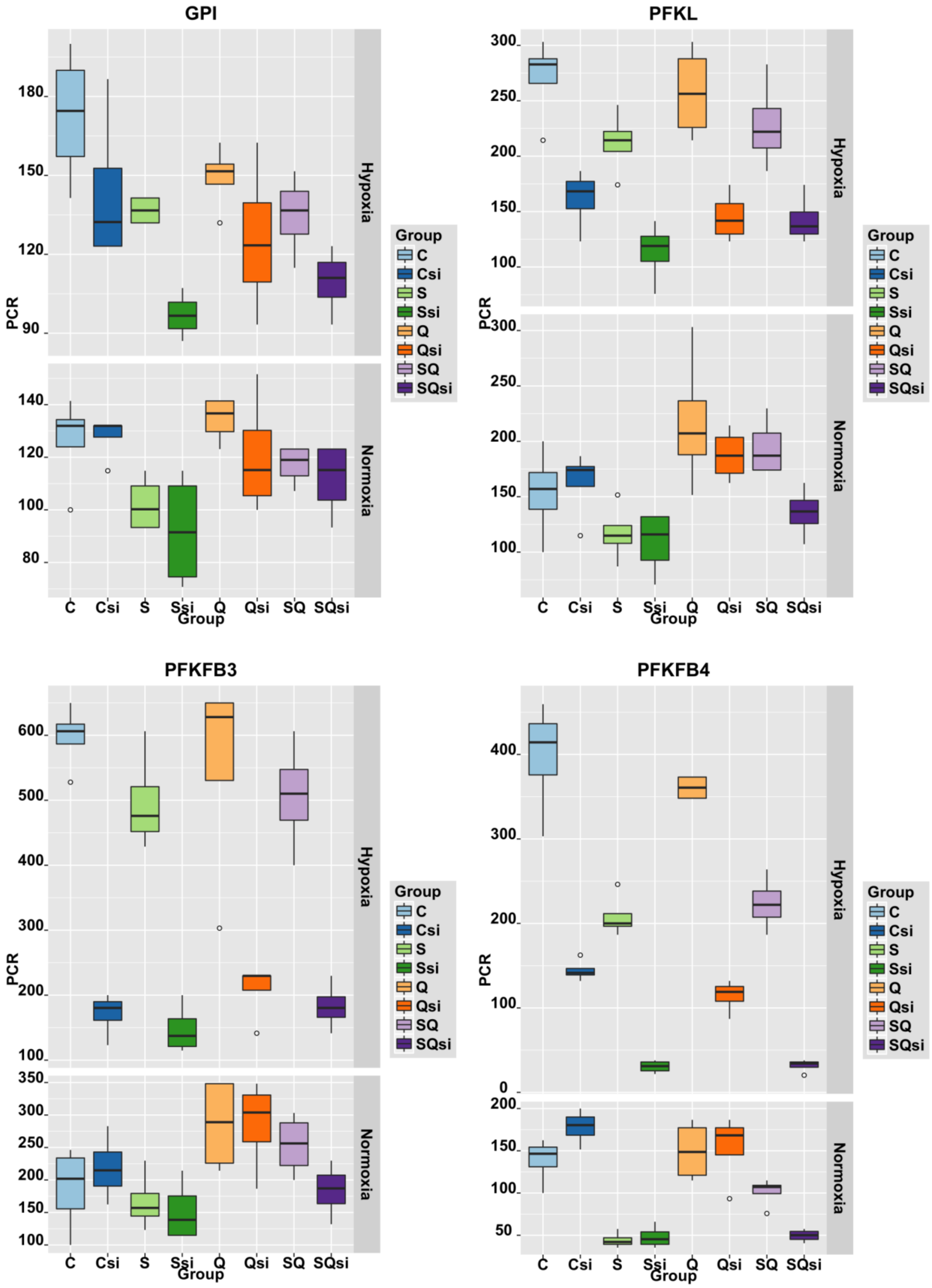
GPI
PFKL
PFKFB3
PFKFB4
PCR
Group
Hypoxia
Normoxia
C
Csi
S
Ssi
Q
Qsi
SQ
SQsi

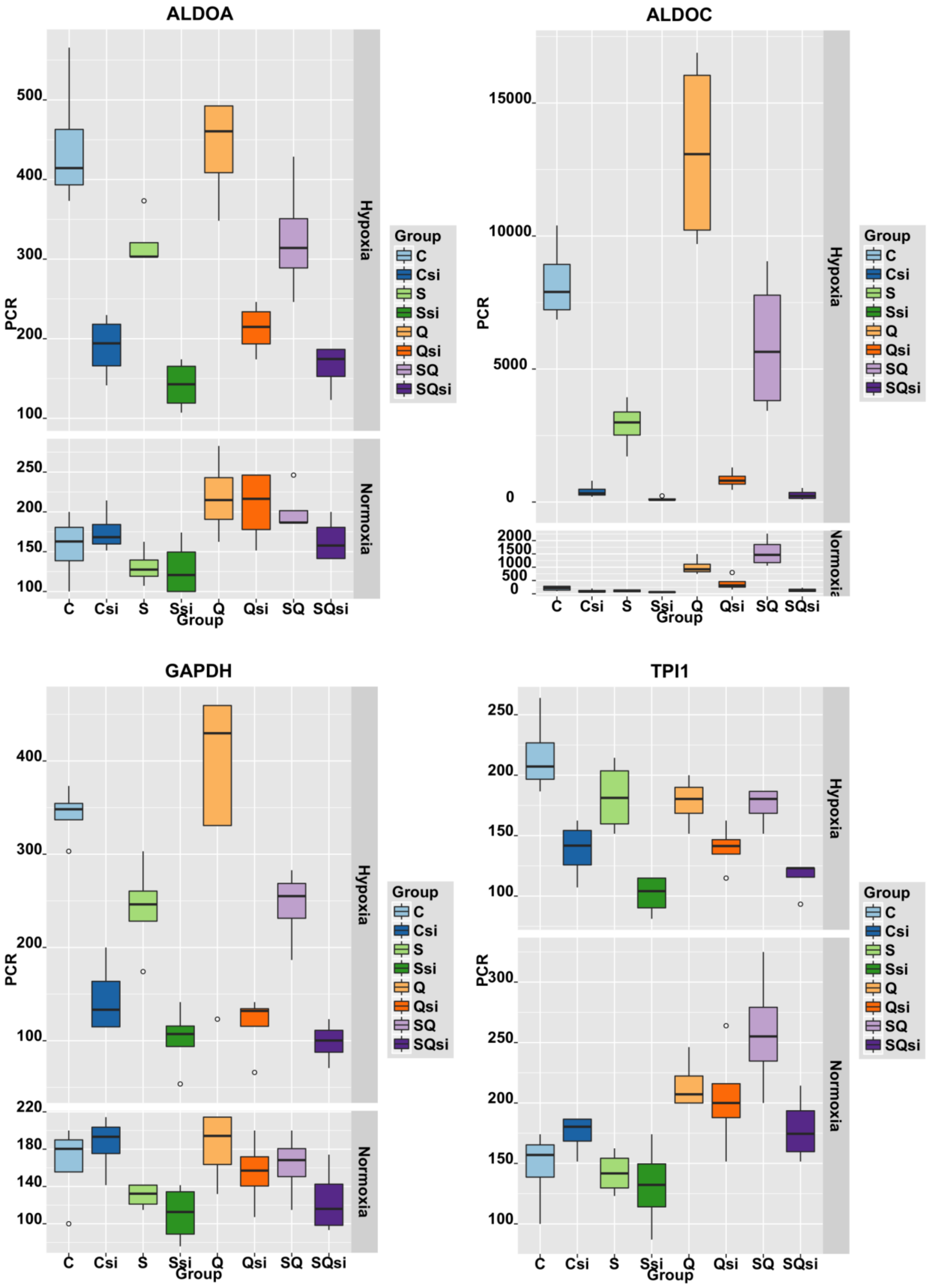
ALDOA
500
400
300
200
100
250
200
150
100
PCR
Hypoxia
Normoxia
C Csi S Ssi Q Qsi SQ SQsi
Group
Group
C
Csi
S
Ssi
Q
Qsi
SQ
SQsi
ALDOC
15000
10000
5000
0
2000
1500
1000
500
0
PCR
Hypoxia
Normoxia
C Csi S Ssi Q Qsi SQ SQsi
Group
Group
C
Csi
S
Ssi
Q
Qsi
SQ
SQsi
GAPDH
400
300
200
100
220
180
140
100
PCR
Hypoxia
Normoxia
C Csi S Ssi Q Qsi SQ SQsi
Group
Group
C
Csi
S
Ssi
Q
Qsi
SQ
SQsi
TPI1
250
200
150
100
300
250
200
150
100
PCR
Hypoxia
Normoxia
C Csi S Ssi Q Qsi SQ SQsi
Group
Group
C
Csi
S
Ssi
Q
Qsi
SQ
SQsi

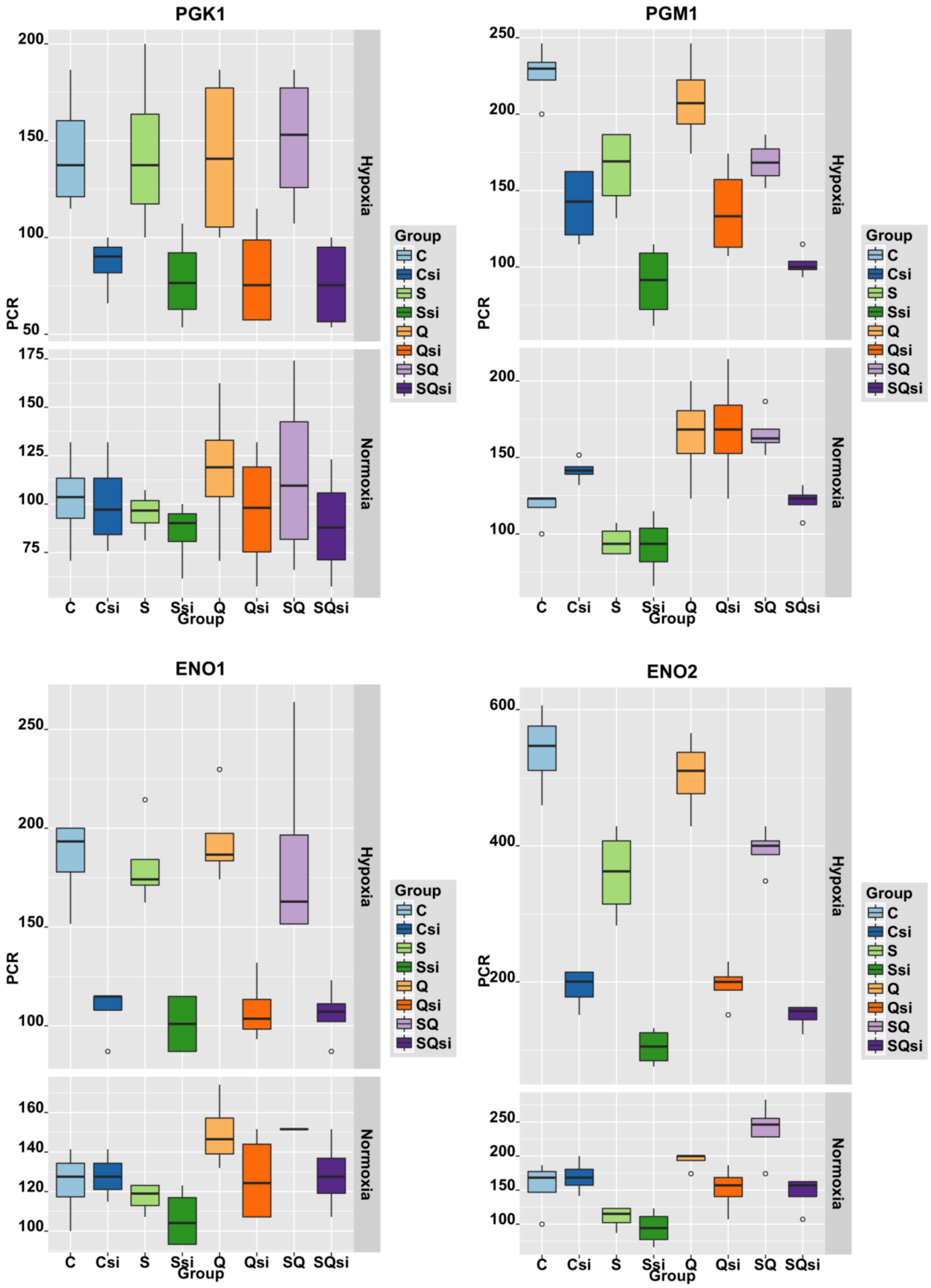
PGK1
PGM1
ENO1
ENO2
PCR
Group
Hypoxia
Normoxia
C
Csi
S
Ssi
Q
Qsi
SQ
SQsi

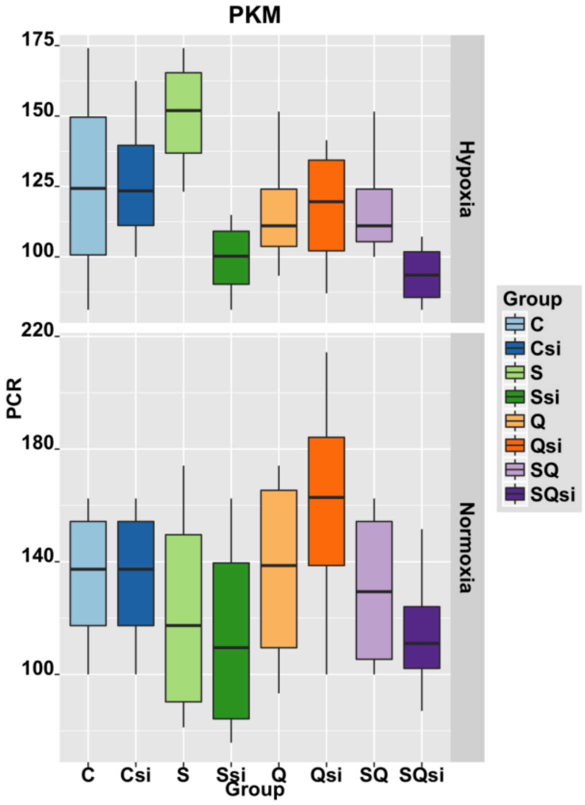
PKM
175
150
125
100
220
180
140
100
PCR
Hypoxia
Normoxia
C Csi S Ssi Q Qsi SQ SQsi
Group
Group
C
Csi
S
Ssi
Q
Qsi
SQ
SQsi

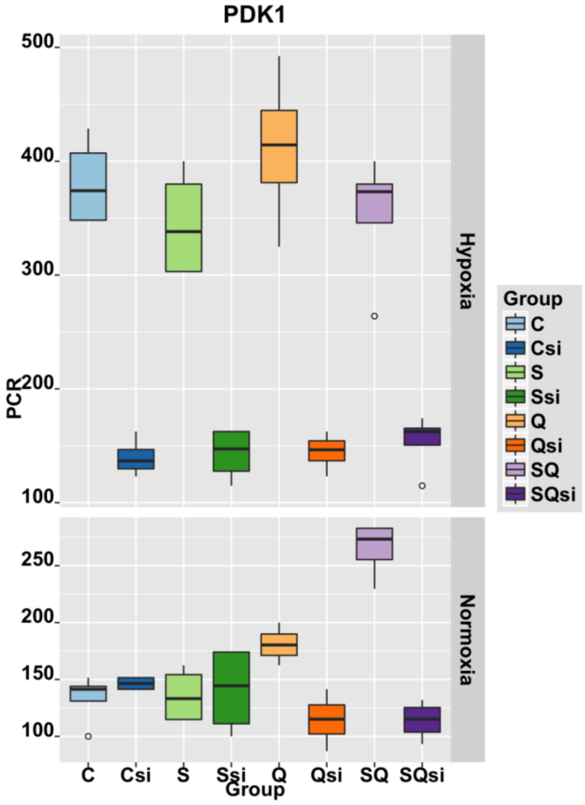
PDK1
500
400
300
200
100
250
200
150
100
PCR
Hypoxia
Normoxia
C Csi S Ssi Q Qsi SQ SQsi
Group
Group
C
Csi
S
Ssi
Q
Qsi
SQ
SQsi

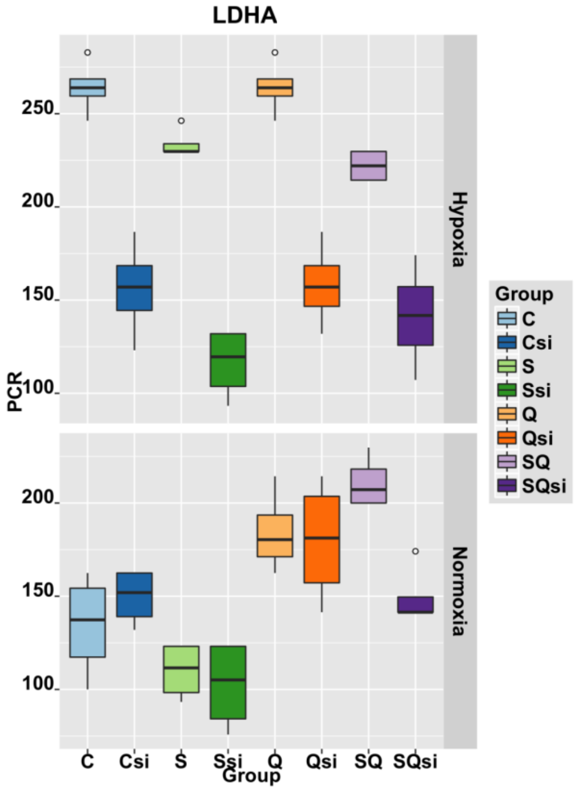
LDHA
250
200
150
100
200
150
100
PCR
Hypoxia
Normoxia
C Csi S Ssi Q Qsi SQ SQsi
Group
Group
C
Csi
S
Ssi
Q
Qsi
SQ
SQsi

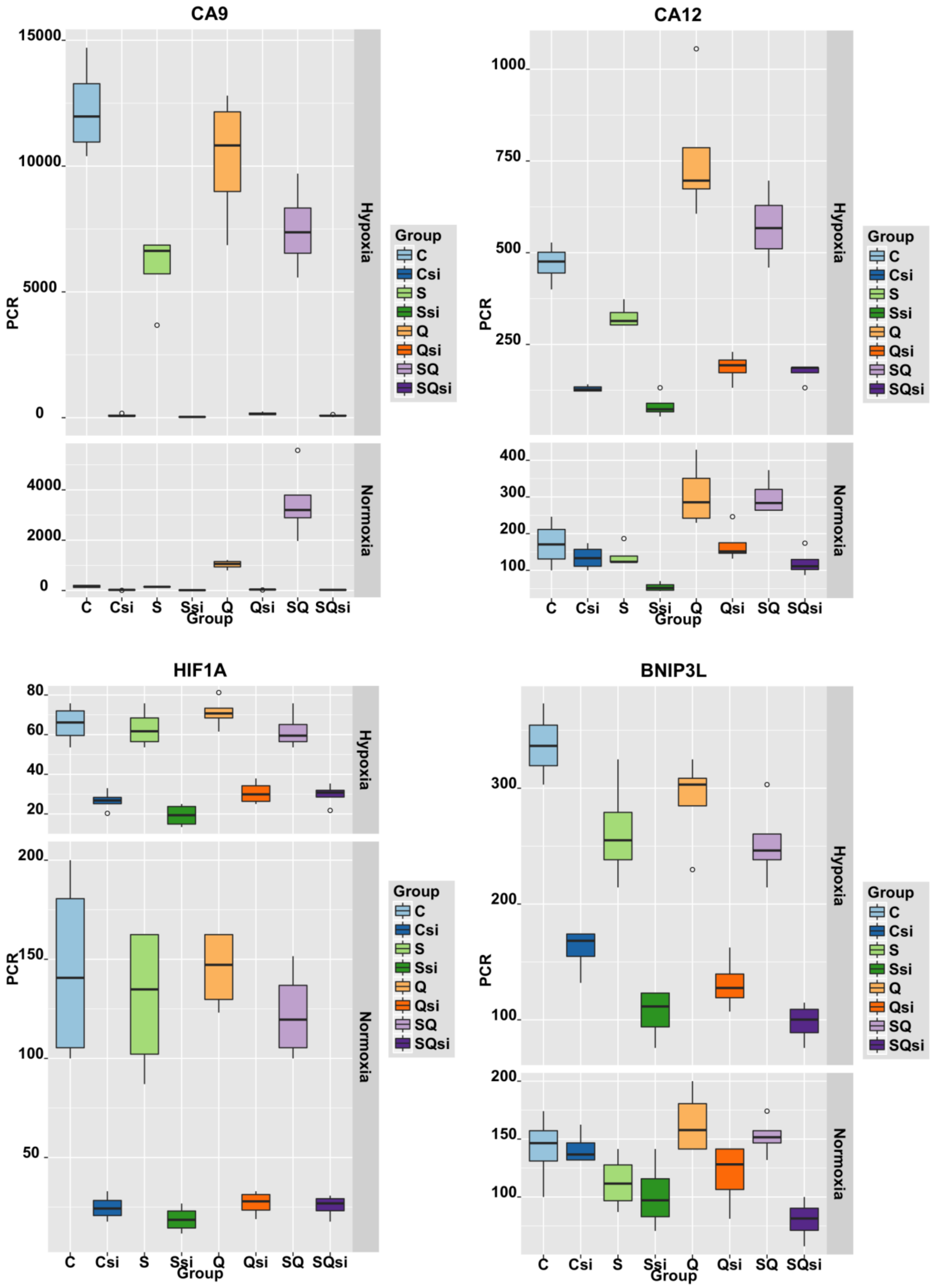

Supplemental Figure S16. Validation of the deep sequencing data by RT-PCR. Relative mRNA level of genes normalized each to RPL9 mRNA level. Application of 5 mM L-glutamine (Q) (lanes 5–8), 1% fetal bovine serum (S) (lanes 2,4,7,8) and/or 5 nM HIF1α-specific siRNA (si) (lanes 2,4,6,8) under normoxic or hypoxic condition in the cell lines MDA-MB-231 24 h after the start of the treatment in four independent experiments.

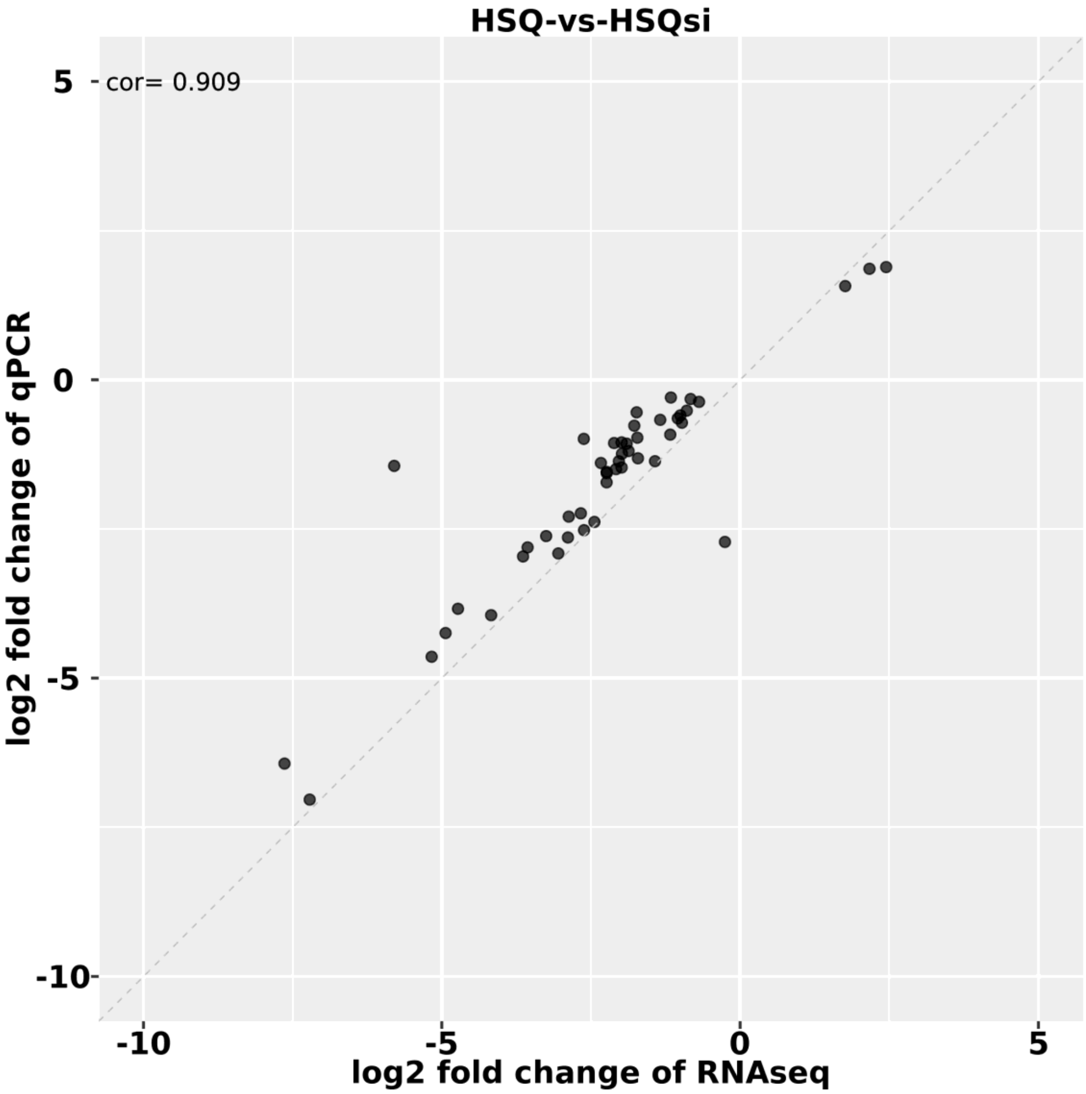

Supplemental Figure S17. Scatterplot of logarithmic fold changes of the deep sequencing analysis compared to the logarithmic fold changes of gene expression due to duplex qPCR for 47 validated genes reaching a correlation of 0.91.

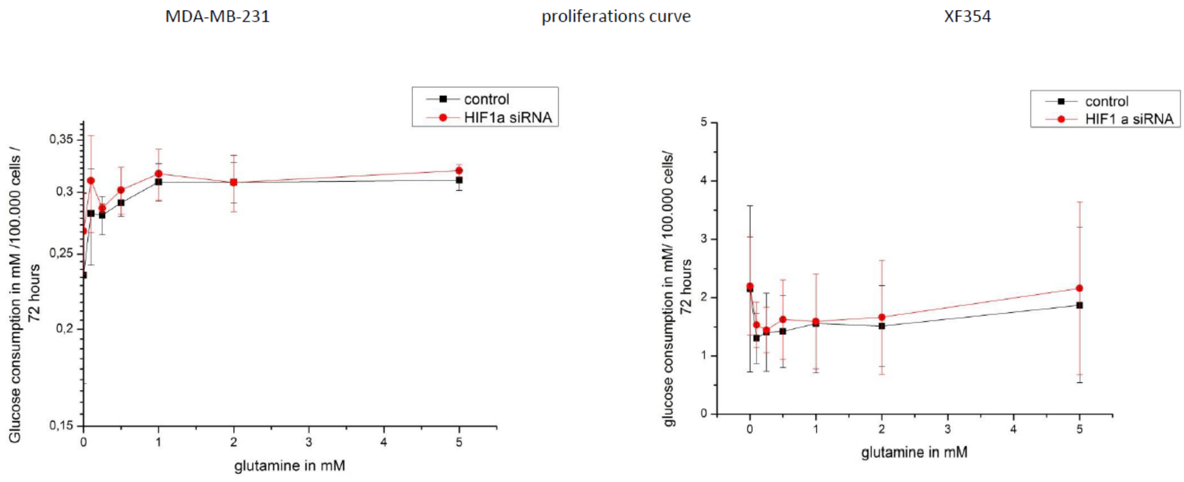

Supplemental Figure S18a. Uptake of glucose was determined for cell lines MDA-MB-231 and XF 354, which were cultivated 72 h with different level of glutamine 0–5 mM in 4 mL medium. The glucose uptake was calculated for MDA-MB-231 cells of approximately 0.3 mM glucose /100,00 cells and for XF354 cells of approximately 1.5 mM 100,000 cell for XF354 cell within 72 h of proliferation,

respectively. The glucose uptake of both cell lines was not affected by different concentrations of glutamine or by HIF1α-specific siRNA. Starting with 11 mM glucose in the media, MDA-MB-231 cells consume a maximum of 7.0 mM and XF 354 cells 7.7 mM glucose.

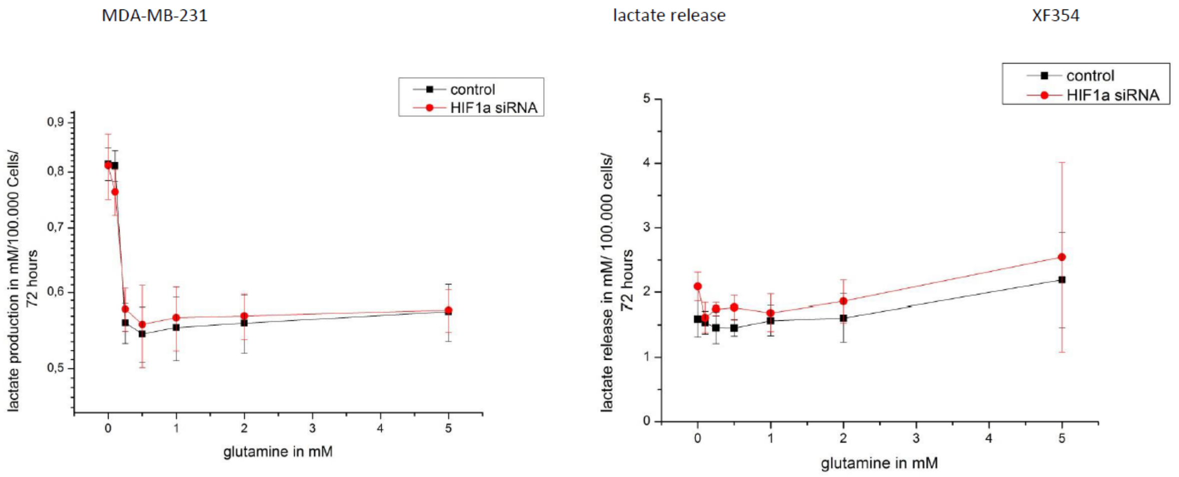

Supplemental Figure S18b. The release of lactate was determined for cell lines MDA-MB-231 and XF 354, which were cultivated 72 h with different level of glutamine 0–5 mM in 4 mL medium. In MDA-MB-231 cells, the relative lactate release was slightly higher in cells cultivated with low concentrations of glutamine (≤0.1 mM glutamine) than in cells cultivated with higher glutamine content. Within 72 h, circa 0.6 mM lactate/100,000 cells were delivered to the medium on average by MDA-MA-231 cells. While the release of lactate of XF354 cells was approximately 2 mM/100,000 cells and was almost constant regarding different glutamine concentrations within 72 h. In the medium of MDA-MB-231 cells, a maximum of 12.7 mM lactate was measured, while in the cell line XF354, a maximum of 11.7 mM lactate after 72 h of cultivation. The lactate concentration of the fresh and unused medium was already removed from the calculations (released lactate), since one lactate source was the serum used in the medium (for the pure serum, a lactate concentration of 15.8 mM was determined).

Moreover, the application of HIF1α-specific siRNA had no effect on the amount of lactate released.

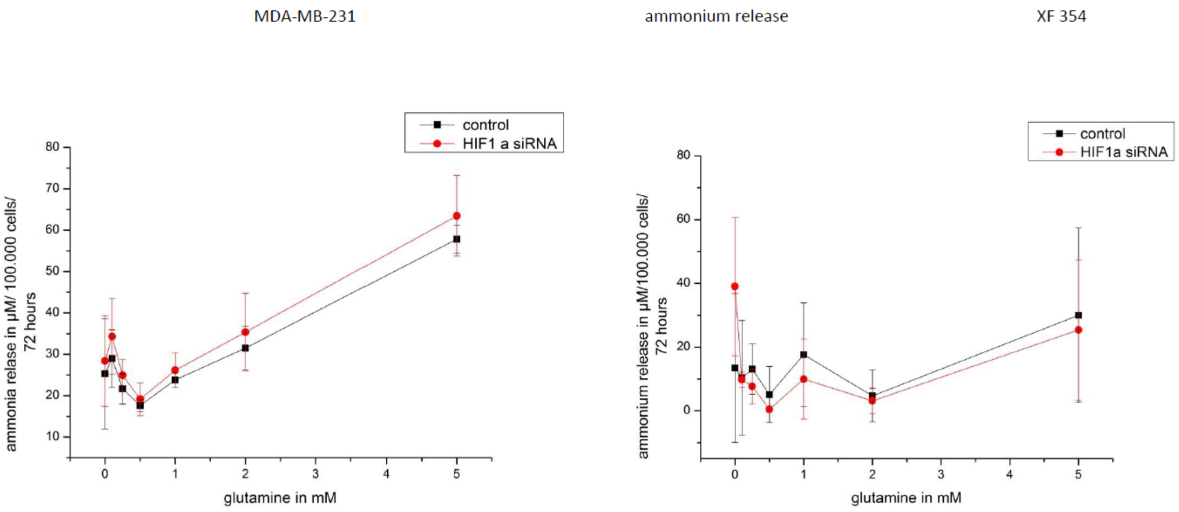

Supplemental Figure S18c. The release of ammonium was determined for cell lines MDA-MB-231 and XF354, which were cultivated 72 h with different level of glutamine 0–5 mM in 4 mL medium. The waste product of the glutamine catabolism, ammonium in the media was analyzed. For MDA-MB-231 cell, the relative ammonium level was around 25 µM/100,00 cell at lower concentrations of glutamine (≤1 mM glutamine) than in cells cultivated with higher glutamine levels >1 mM, where the ammonium level increases to 60 µm/100,000 within 72 h at 5 mM glutamine. The release of ammonium of XF354 cells was in a range between 10−20 µM/100,000 cells within 72 h. After 72 h of

cultivation of MDA-MB-231 cells, approximately 1.7 mM ammonium was detected using 5 mM glutamine. At a level of 2 mM glutamine (most cell culture media contain such an amount of glutamine) we were still able to detect nearly 1 mM ammonium after 72 h of cultivation in the media. After 72 h of cultivation the minimal ammonium concentration in the medium was at least 100 µM. In the cell line XF354, a maximum of 500 µM ammonium after 72 h of cultivation was found. The ammonium concentration of the unused medium (decay of glutamine) was already removed for the calculations of the ammonium concentration released by the cell (Supplemental Figure 21). The most important source of ammonium was the used glutamine solution (200 mM glutamine contained about 3.4 mM ammonium per se) and the used serum in the medium contained 700 µM ammonium. The application of HIF1α-specific siRNA had no effect on the amount of ammonium released. In a pilot study the used medium was analyzed via nuclear magnetic resonance which identified alanine as deregulated substance.

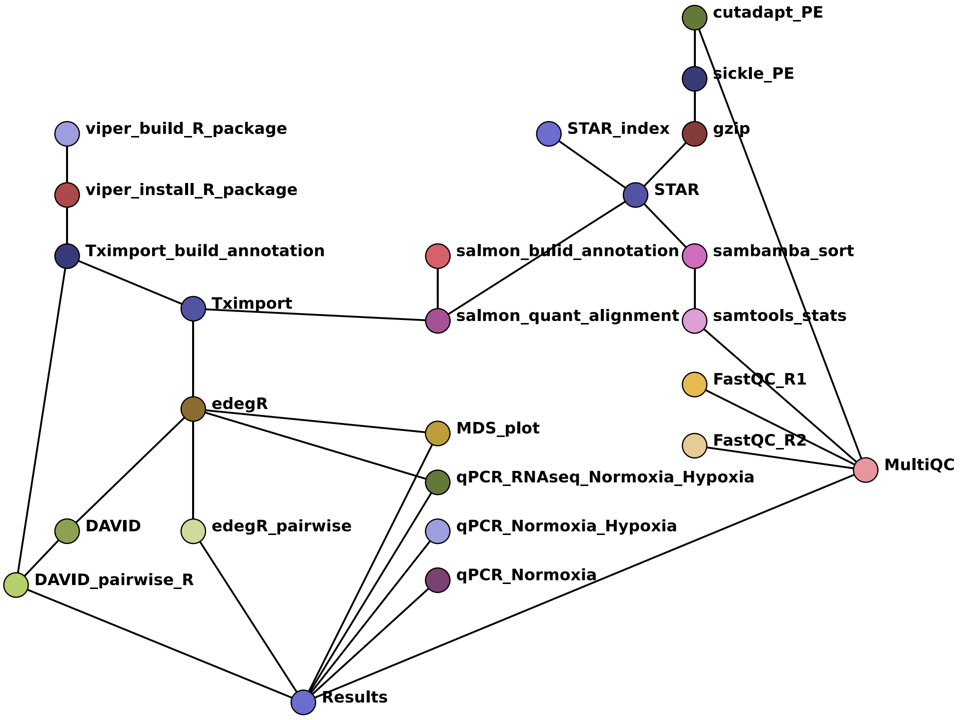

Supplemental Figure S19. The workflow performs differential expression analysis on paired-end RNA-seq data and was build with the workflow management system snakemake. After adapter removal with Cutadapt and quality filtering with sickle, reads were mapped with STAR to the humane genome (GRCh38.82), and transcript counts were quantified with salmon. These transcript counts were summarized to gene counts with tximport. A gene is defined as expressed if it exhibited more than one counts in at least two out of four biological replicates in at least one treatment. Genes which were not expressed according to our definition were removed from the analysis. Hence, we also kept genes in the analysis showing expression in just one treatment. Integrated normalization and differential expression analysis were conducted with edgeR for all expressed genes. The genes were declared as differentially expressed if the adjusted false discovery rate (FDR)was below 0.05, and the log2-fold change was greater than one or smaller than minus one. Further, we used the Database for Annotation, Visualization and Integrated Discovery (DAVID v6_8) for functional annotation of the differential expressed genes. See also https://github.com/GrosseLab/VipeR_HIF1alpha/, last accessed on: 16 September 2019.

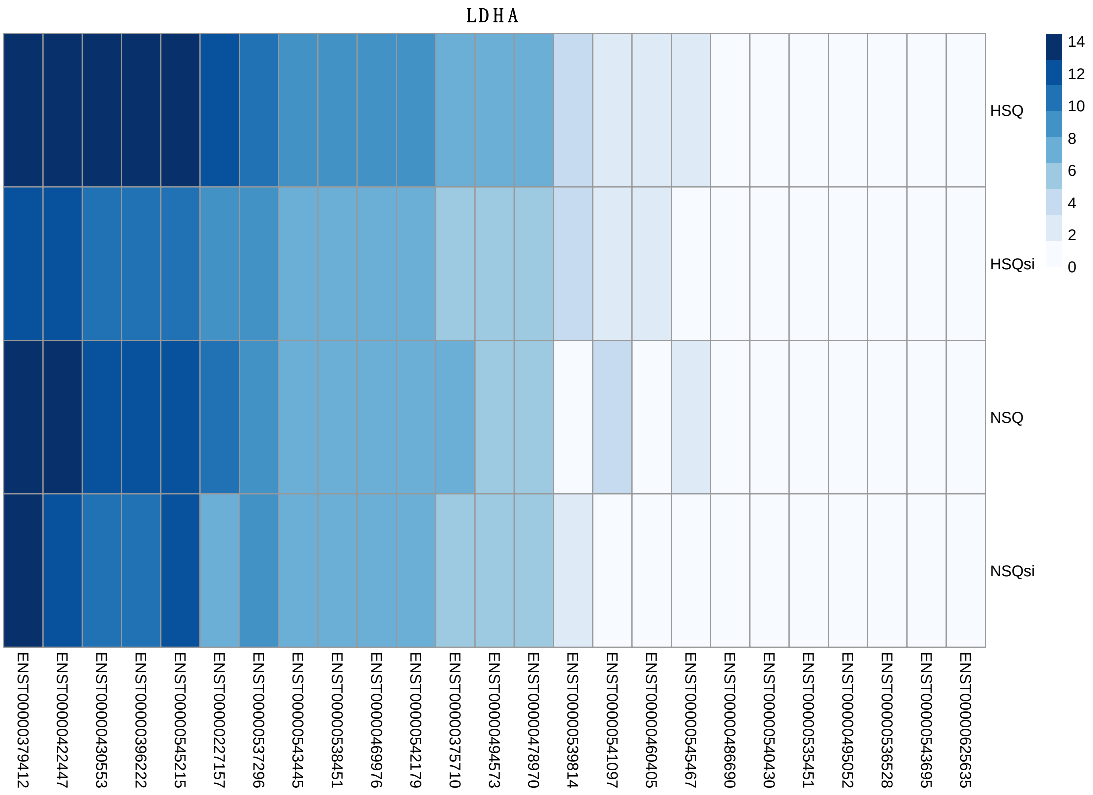

Supplemental Figure S20. Example of LDHA splice variants. Interestingly, the LDHA mRNA level measured with salmon is induced by HIF1 for only 14 splice variants of LDHA (average logarithmic expression > 5). The average logarithmic expressions for each condition are plotted in blue.

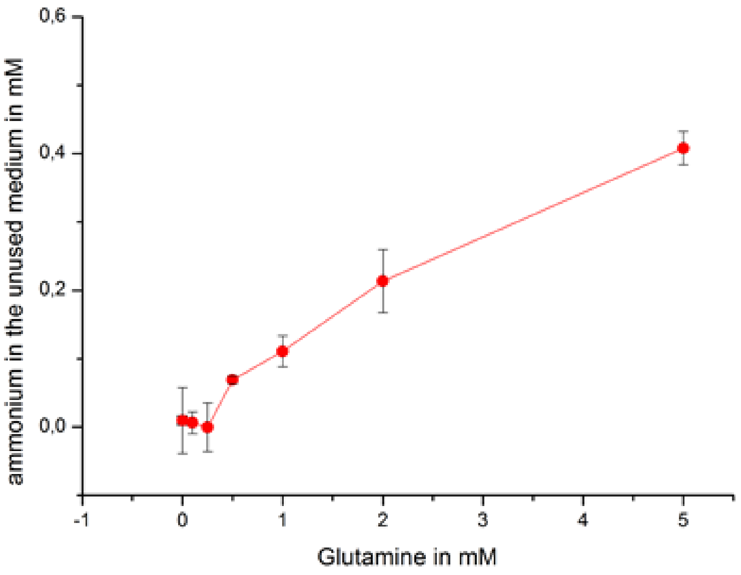

Supplemental Figure S21. Decay glutamine in the unused medium by analyzing the ammonium level. Since glutamine is decayed into glutamate and ammonium the corresponding medium of the experiments was also analyzed. The ammonium release due to the decay of the used amino acid glutamine was <100 µM ammonium using glutamine levels of <0.5 mM and increased with higher glutamine levels (0.5 mMQ≙69 µM ± 6 µM ammonium; 1 mMQ≙110 µM ± 20 µM ammonium; 2 mMQ≙210 µM ± 46 µM ammonium; 5 mMQ≙410 µM ± 20 µM ammonium). The ammonium concentration of the unused medium (decay of glutamine) was already removed for the calculations

of the ammonium concentration released by the cell, of course (for Supplemental Figure S18c). The most important source of ammonium was the used glutamine solution (200 mM glutamine contained about 3.4 mM ammonium per se) and the used serum in the medium contained 700 µM ammonium.

Supplemental Table S1. Deep sequencing data of HIF1α/CAIX positive samples (Figure1 lane 7 and 7*) treated with 1) 5 mM glutamine and 1% fetal bovine serum and HIF1α/ CAIX negative samples positive samples are visualised as (Figure1 lane 8 and 8*) of the investigated carcinoma cell line MDA-MB-231 were treated identically with 2) 5 mM glutamine and 1% fetal bovine serum, but they were also additionally treated with HIF1α-specific siRNA 24 h before the experiment was started. (green marked genes were significantly regulated by HIF1).

		HYPOXIA				NORMOXIA			
Gensymbol	Ensembl nummer	log2TMM noSi	log2TMM Si	log2fold	FDR	log2TMM noSi	log2TMM Si	log2fold	FDR
SLC2A1	ENSG00000117394	14,71	12,73	-1,98	8.5e-95	13,47	12,5	-0,97	3.8e-14
SLC2A3	ENSG00000059804	14,23	12,8	-1,43	8.6e-29	11,15	11,7	0,55	9.6e-06
HK1	ENSG00000156515	13,56	12,51	-1,04	9.6e-16	12,45	11,86	-0,59	2.4e-09
HK2	ENSG00000159399	14,72	12,82	-1,9	9.2e-79	13,23	11,96	-1,27	1.6e-47
GPI	ENSG00000105220	14,68	13,52	-1,16	4.7e-23	14,06	13,69	-0,37	7.5e-03
PFKL	ENSG00000141959	12,34	11	-1,34	2.7e-23	11,85	11,11	-0,75	6.6e-07
PFKM	ENSG00000152556	11,63	11,83	0,2	1.1e-01	11,46	11,28	-0,17	6.9e-01
PFKP	ENSG00000067057	14,22	13,44	-0,78	4.9e-12	13,72	13,4	-0,32	6.6e-03
PFKFB1	ENSG00000158571	0,75	0,67	-0,09	1.0e+00	0,6	-1	-1,6	8.6e-01
PFKFB2	ENSG00000123836	9,63	10,14	0,51	8.2e-06	9,75	9,91	0,16	5.9e-01
PFKFB3	ENSG00000170525	13,4	11,41	-1,99	1.4e-90	12,11	11,44	-0,67	6.1e-11
PFKFB4	ENSG00000114268	12,25	8,69	-3,56	1.3e-109	10,64	9,49	-1,15	1.7e-17
ALDOA	ENSG00000149925	16,08	14,35	-1,72	5.8e-33	14,9	14,38	-0,53	1.5e-04
ALDOC	ENSG00000109107	8,9	3,73	-5,17	7.7e-84	6,11	2,67	-3,44	8.5e-12
TPI1	ENSG00000111669	15,23	14,23	-1	3.8e-31	15,2	14,58	-0,62	4.1e-06
GAPDH	ENSG00000111640	17,97	16,26	-1,71	1.8e-57	17,1	16,57	-0,54	1.1e-04
PGK	ENSG00000102144	17,08	14,46	-2,62	3.1e-114	15,88	14,63	-1,25	4.0e-24
PGM1	ENSG00000079739	12,17	11,19	-0,98	1.2e-25	11,97	11,33	-0,64	6.0e-08
PGM2	ENSG00000169299	11,77	11,65	-0,12	4.6e-01	11,8	11,62	-0,18	3.4e-01
PGM3	ENSG00000013375	10,92	10,73	-0,19	2.7e-01	10,88	10,83	-0,05	9.9e-01
ENO1	ENSG00000074800	16,81	15,04	-1,77	2.5e-72	16,22	15,52	-0,7	9.5e-10
ENO2	ENSG00000111674	13,83	11,49	-2,33	7.7e-95	12,35	11,14	-1,22	5.0e-33
ENO3	ENSG00000108515	9,69	9,33	-0,36	1.6e-02	8,74	8,51	-0,23	4.8e-01
PKM	ENSG00000067225	16,66	15,83	-0,83	5.6e-22	16,45	16,16	-0,3	2.3e-02
PDHA1	ENSG00000131828	11,61	12,08	0,48	7.9e-07	11,97	12,21	0,24	1.2e-01
PDK1	ENSG00000152256	12,34	10,47	-1,87	2.1e-59	11,54	9,6	-1,95	1.8e-57
PDK2	ENSG00000005882	8,01	8,36	0,35	5.9e-02	8,71	9,08	0,37	1.6e-01
PDK3	ENSG00000067992	10,04	9,02	-1,02	1.1e-13	9,52	8,95	-0,57	7.2e-03
PDK4	ENSG00000004799	2,98	3,52	0,55	5.8e-01	4,12	3,46	-0,66	8.9e-01
LDHA	ENSG00000134333	16,04	14,31	-1,73	7.1e-63	15,44	14,33	-1,11	1.2e-18
SLC16A3	ENSG00000141526	9,91	8,9	-1	3.7e-05	9,33	9,14	-0,19	8.1e-01
Control									
HIF1A	ENSG00000100644	11,3	9,32	-1,99	4.0e-54	12,75	8,94	-3,82	5.7e-211
CA9	ENSG00000107159	9,54	1,91	-7,64	3.9e-170	8,19	-1,35	-9,54	3.2e-45
RPL9	ENSG00000163682	13,82	13,72	-0,1	5.1e-01	13,53	13,62	0,09	8.8e-01

Supplemental Table S2. Normalised qPCR expressions values for normoxic and hypoxic treatments are fitted gene wise with a linear model with three factors.

Gensymbol	Name	Emsembl nummer	HYPOXIA (siRNA)	NORMOXIA (siRNA and Q)
SLC2A1	solute carrier family 2 (facilitated g	ENSG00000117394	p<0.001	p=0.18
SLC2A3	solute carrier family 2 (facilitated g	ENSG00000059804	p=0.008	p=0.39
HK1	hexokinase 1 [Source:HGNC Symbo	ENSG00000156515	p<0.001	p=0.09
HK2	hexokinase 2 [Source:HGNC Symbo	ENSG00000159399	p<0.001	p<0.001
GPI	glucose-6-phosphate isomerase [S	ENSG00000105220	p<0.001	p=0.62
PFKL	phosphofructokinase; liver [Source	ENSG00000141959	p<0.001	p=0.09
PFKFB3	6-phosphofructo-2-kinase/fructose	ENSG00000170525	p<0.001	p=0.30
PFKFB4	6-phosphofructo-2-kinase/fructose	ENSG00000114268	p<0.001	p=0.016
ALDOA	aldolase A; fructose-bisphosphate	ENSG00000149925	p<0.001	p=0.22
ALDOC	aldolase C; fructose-bisphosphate	ENSG00000109107	p<0.001	p<0.001
TPI1	triosephosphate isomerase 1 [Sour	ENSG00000111669	p<0.001	p=0.032
GAPDH	glyceraldehyde-3-phosphate dehy	ENSG00000111640	p<0.001	p=0.19
PGK1	phosphoglycerate kinase 1 [Source	ENSG00000102144	p<0.001	p=0.42
PGM1	phosphoglucomutase 1 [Source:HG	ENSG00000079739	p<0.001	p=0.047
ENO1	enolase 1; (alpha) [Source:HGNC Sy	ENSG00000074800	p<0.001	p=0.09
ENO2	enolase 2 (gamma; neuronal) [Sour	ENSG00000111674	p<0.001	p=0.005
PDK1	pyruvate dehydrogenase kinase; is	ENSG00000152256	p<0.001	p<0.001
LDHA	lactate dehydrogenase A [Source:H	ENSG00000134333	p<0.001	p=0.027
Control				
HIF1A	hypoxia inducible factor 1; alpha su	ENSG00000100644	p<0.001	p=0.65
CA9	carbonic anhydrase IX [Source:HGN	ENSG00000107159	p<0.001	p<0.001
CA12	carbonic anhydrase XII [Source:HGN	ENSG00000074410	p<0.001	p=0.01
BNIP3L	BCL2/adenovius E1B 19kDa interact	ENSG00000104765	p<0.001	p<0.001

Supplemental Table S3. Transfection diagram. *according to the manufacturers' instructions.

	siRNA		Transfection	
	**siRNA concentration**	siRNA (origin)	time	Transfection reagent
HIF1α-siRNA	5 nM or 10nM	Silencer®Select Validated siRNA ID: s6539 (Life Technologies, Darmstadt, Germany)	overnight	*INTERFERin reagent (Polyplus Transfection, Illkirch, France) without glutamine, normoxic, 0%FBKS in RPMI 1640
ACLY-siRNA	20 nM	Silencer®Select Validated siRNA ID: s916 (Life Technologies, Darmstadt, Germany)	48 h	*INTERFERin reagent (Polyplus Transfection, Illkirch, France) without glutamine, normoxic, 1%FBKS in RPMI 1640
ACSS2-siRNA	20 nM	Silencer® Select Pre-Designed siRNA siRNA ID: s31746 (Life Technologies, Darmstadt, Germany)	48 h	*INTERFERin reagent (Polyplus Transfection, Illkirch, France) without glutamine, normoxic 1%FBS RPMI 1640
